# Human milk antibodies to global pathogens reveal geographic and interindividual variations in IgA and IgG

**DOI:** 10.1172/JCI168789

**Published:** 2024-06-11

**Authors:** Joseph J. Campo, Antti E. Seppo, Arlo Z. Randall, Jozelyn Pablo, Chris Hung, Andy Teng, Adam D. Shandling, Johnathon Truong, Amit Oberai, James Miller, Najeeha Talat Iqbal, Pablo Peñataro Yori, Anna Kaarina Kukkonen, Mikael Kuitunen, L. Beryl Guterman, Shaun K. Morris, Lisa G. Pell, Abdullah Al Mahmud, Girija Ramakrishan, Eva Heinz, Beth D. Kirkpatrick, Abu S.G. Faruque, Rashidul Haque, R. John Looney, Margaret N. Kosek, Erkki Savilahti, Saad B. Omer, Daniel E. Roth, William A. Petri, Kirsi M. Järvinen

**Affiliations:** 1 Antigen Discovery Incorporated, Irvine, California, USA.; 2 Department of Pediatrics, Division of Allergy and Immunology, University of Rochester School of Medicine, Rochester, New York, USA.; 3 Department of Paediatrics and Child Health, Biological and Biomedical Sciences, Aga Khan University, Karachi, Pakistan.; 4 Division of Infectious Diseases and International Health, University of Virginia, Charlottesville, Virginia, USA.; 5 New Children’s Hospital, University of Helsinki and Helsinki University Hospital, Helsinki, Finland.; 6 Hubert Department of Global Health, Rollins School of Public Health, Emory University, Atlanta, Georgia, USA.; 7 Centre for Global Child Health, Hospital for Sick Children, Toronto, Ontario, Canada.; 8 Department of Pediatrics, University of Toronto, Hospital for Sick Children, Toronto, Ontario, Canada.; 9 Nutrition and Clinical Services Division, International Centre for Diarrhoeal Disease Research, Dhaka, Bangladesh.; 10 Departments of Vector Biology and Clinical Sciences, Liverpool School of Tropical Medicine, Liverpool, UK.; 11 Wellcome Sanger Institute, Parasites and Microbes, Cambridge, UK.; 12 Vaccine Testing Center and Department of Microbiology and Molecular Genetics, The University of Vermont College of Medicine, Burlington, Vermont, USA.; 13 Emerging Infection and Parasitology Laboratory, Division of Infectious Diseases, International Centre for Diarrhoeal Disease Research, Dhaka, Bangladesh.; 14 Department of Medicine, Division of Allergy, Immunology and Rheumatology, University of Rochester School of Medicine, Rochester, New York, USA.; 15 Peter O’Donnell Jr. School of Public Health, Dallas, Texas, USA.; 16 Department of Microbiology and Immunology, University of Rochester School of Medicine, Rochester, New York, USA.

**Keywords:** Infectious disease, Adaptive immunity, Immunoglobulins

## Abstract

**BACKGROUND:**

The use of high-throughput technologies has enabled rapid advancement in the knowledge of host immune responses to pathogens. Our objective was to compare the repertoire, protection, and maternal factors associated with human milk antibodies to infectious pathogens in different economic and geographic locations.

**METHODS:**

Using multipathogen protein microarrays, 878 milk and 94 paired serum samples collected from 695 women in 5 high and low-to-middle income countries (Bangladesh, Finland, Peru, Pakistan, and the United States) were assessed for specific IgA and IgG antibodies to 1,607 proteins from 30 enteric, respiratory, and bloodborne pathogens.

**RESULTS:**

The antibody coverage across enteric and respiratory pathogens was highest in Bangladeshi and Pakistani cohorts and lowest in the U.S. and Finland. While some pathogens induced a dominant IgA response (*Campylobacter*, *Klebsiella,* *Acinetobacter*, *Cryptosporidium,* and pertussis), others elicited both IgA and IgG antibodies in milk and serum, possibly related to the invasiveness of the infection (*Shigella,* enteropathogenic *E*. *coli* “EPEC”*, Streptococcus pneumoniae, Staphylococcus aureus,* and Group B *Streptococcus)*. Besides the differences between economic regions and decreases in concentrations over time, human milk IgA and IgG antibody concentrations were lower in mothers with high BMI and higher parity, respectively. In Bangladeshi infants, a higher specific IgA concentration in human milk was associated with delayed time to rotavirus infection, implying protective properties of antirotavirus antibodies, whereas a higher IgA antibody concentration was associated with greater incidence of *Campylobacter* infection.

**CONCLUSION:**

This comprehensive assessment of human milk antibody profiles may be used to guide the development of passive protection strategies against infant morbidity and mortality.

**FUNDING:**

Bill and Melinda Gates Foundation grant OPP1172222 (to KMJ); Bill and Melinda Gates Foundation grant OPP1066764 funded the MDIG trial (to DER); University of Rochester CTSI and Environmental Health Sciences Center funded the Rochester Lifestyle study (to RJL); and R01 AI043596 funded PROVIDE (to WAP).

## Abstract 2

Multipathogen protein microarray analysis of milk and serum samples from women in high- and low/middle-income countries reveals geographic and interindividual variations in anti-pathogen antibodies.

## Graphical abstract

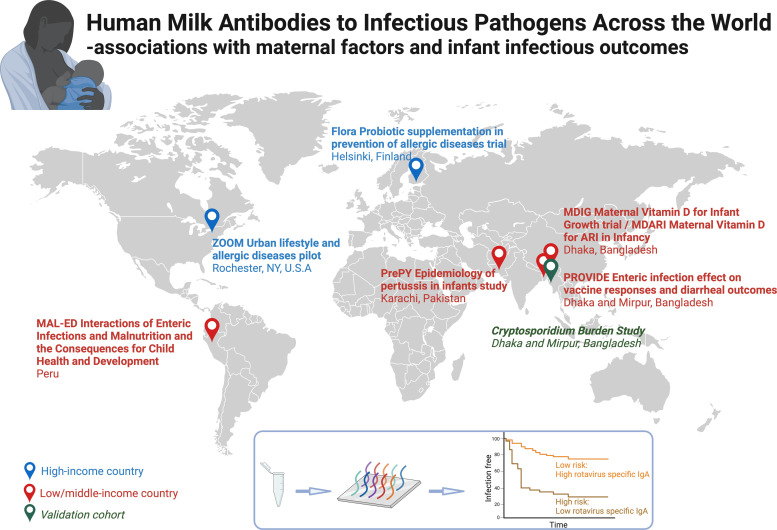

## Introduction

In 2019, 5.3 million liveborn children died before 5 years of age worldwide, nearly half of whom died from infectious diseases, including lower respiratory infections and diarrhea (1). The enteric pathogens causing the most morbidity and mortality in developing countries throughout Asia, Africa, and South America include *Shigella*, heat-stable and heat-labile enterotoxigenic *E*. *coli* (ETEC)*,* typical enteropathogenic *E*. *coli* (EPEC), *Cryptosporidium*, *Campylobacter jejuni/Campylobacter coli,* rotavirus, adenovirus 40/41, norovirus GII, and astrovirus (2, 3). Microorganisms associated with pneumonia in children under 5 years old in Asia, South America, and Africa include *Streptococcus pneumoniae*, respiratory syncytial virus (RSV), rhinovirus, human metapneumovirus, influenza A, parainfluenza 1/3, and influenza B, adenovirus, and enterovirus (4). Lastly, about a third of neonatal sepsis cases globally were culture confirmed, and the most common pathogens overall were *S*. *aureus* and *Klebsiella* spp. (5). Although the Millennium Development Goal (MDG) four, to reduce child mortality by two-thirds between 1990 and 2015 was not met, there was over 50% decrease worldwide in mortality in children under 5 years, in part attributed to infant vaccinations (1). Breastfeeding has been shown to reduce infant mortality, specifically from diarrhea and acute respiratory infections (6–14), and potentially also neonatal sepsis (15, 16). Because the protection is likely, at least in part, due to human milk antibodies, boosting the passive protection provided to the infant by human milk antibodies could provide an additional strategy to further decrease infant morbidity and mortality.

Human milk is a rich source of immunomodulatory factors including antibodies, oligosaccharides, and antimicrobial peptides that may play a role in protection against infectious diseases in breastfed infants. The predominant immunoglobulin in human milk is IgA, most of which is secretory IgA (SIgA), with smaller amounts of IgG and IgM. Higher colostrum and mature milk antibody concentrations against enteric pathogens have been associated with protection against diarrheal diseases caused by rotavirus (17, 18), enterovirus (19), Shigella (20), *Giardia* lamblia (21, 22), *Entamoeba histolytica,* and *Cryptosporidium* (23) and *Campylobacter* (24–26). Regarding respiratory pathogens, prior studies have focused on the effect of maternal vaccination during pregnancy on human milk concentrations of IgG and IgA to pertussis, pneumococcus, influenza, and meningococcus (27), with limited assessment of the impact of these antibodies on rates of specific infections in breastfed children. Vaccinated Bangladeshi mothers have significantly higher specific, viral neutralizing IgA concentrations to influenza A/New Caledonia in human milk compared with those not vaccinated (28). Further, exclusive breastfeeding in the first 6 months of life significantly reduced the number of infant febrile respiratory illnesses, which implies protective properties of these milk antibodies (28). Lastly, human milk levels of capsular antibodies to Group B *Streptococcus* (GBS) were associated with decreased risk of late-onset neonatal infections (29). However, few reports of large-scale profiling of antibody responses in maternal milk and serum exist to uncover possible gaps that might explain the disparities in the morbidity and mortality from infectious diseases across high-income countries (HICs) and low-and-middle-income countries (LMICs).

Antibody profiling for multiple pathogens has been limited in the past due to the complexity of each pathogen and lack of available multiplex tools, with the majority of past studies assessing single pathogens with ELISA. The use of high-throughput technologies like proteome microarrays has enabled rapid advancement in knowledge of host immune responses to such complex pathogens. We recently used high-throughput expressible open reading frame (ORF) libraries for infectious organisms and exploited in vitro transcription and translation (IVTT) for production of proteomic microarray chips to characterize IgA and IgG specificity in human milk. This unique high-throughput cloning and expression platform enables the construction of protein microarrays for any organism whose genome sequence has been determined and where a source of genomic DNA is available. We assessed human milk antibodies to multiple pathogens known to cause infantile enteric and respiratory diseases in LMICs, such as enteroaggregative *Escherichia coli* (EAEC), EPEC, ETEC, *Shigella, Salmonella enterica* serovar Typhi, *Streptococcus pneumoniae, Staphylococcus aureus*, *Mycobacterium tuberculosis (Mtb),* influenza, RSV, measles, rubella and pertussis among others (30). We found that human milk IgA to EPEC and *Shigella* antigens were higher in LMICs than in Europe and the northwest region of the U.S.

In the present study, these protein arrays were expanded to include additional pathogen antigens (*Campylobacter jejuni*, *Cryptosporidium parvum* and *hominis*, *Vibrio cholerae*, rotavirus, adenovirus, *Klebsiella pneumoniae*, GBS, and *Acinetobacter baumannii*) associated with substantial infant morbidity and mortality across the world. The expanded protein arrays were used to assess fundamental questions regarding the coverage and concentrations of human milk antibodies in breastfeeding women from HICs and LMICs, the differences between maternal systemic (serum) and mucosal (human milk) antibody repertoires, the effect on antibody concentrations by maternal factors, and the possible protective properties of human milk antibodies against infant infectious outcomes. These studies are instrumental in designing strategies to boost maternal antibody concentrations or to adequately supplement human milk or human milk alternatives for the benefit of infant health.

## Results

### Multipathogen protein microarray, cohort, and sample characteristics.

**Figure 1:**
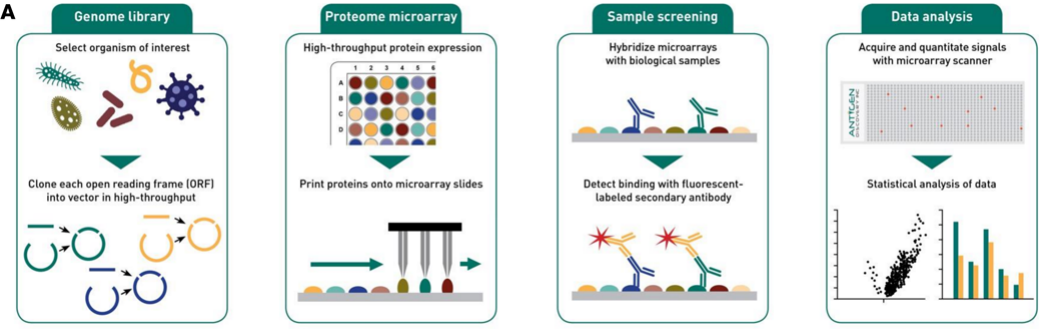
Multipathogen protein microarray principle. Open reading frame (ORF) expression clone libraries can be constructed from any genome sequence and corresponding source of genomic DNA using high-throughput PCR/recombination cloning. Proteins encoded by the cloned ORF plasmids are expressed using a cell-free in vitro transcription/translation (“IVTT”) system. Each protein is expressed and printed individually onto microarray slides. With as little as 2–5 μL of serum and 100 μL of defatted human milk, complete or partial proteomes can be screened for antibody binding. Isotype-specific bound antibodies are detected using a fluorescently labeled secondary antibody. Using a fluorescence microarray scanner, signal intensities from protein microarrays are acquired and checked for quality, followed by statistical analysis.

The multipathogen array was developed at Antigen Discovery Inc. using an established high throughput cloning and protein expression system (Figure 1). Arrays were designed to include approximately 80 proteins from each of the enteric, respiratory, and sepsis-related pathogens responsible for most morbidity and mortality from infectious diseases in children under 5 years, as well as 5–15 proteins from other pathogens relevant to global health. Selection of proteins followed 2 approaches: (a) an empirical approach utilizing the databases from prior studies performed at Antigen Discovery Inc., and (b) a hypothetical approach using in silico prediction of antigenic targets and orthologues of confirmed antigenic targets already identified in ADI databases. Proteins were selected for inclusion based on seroprevalence rates and correlation with exposure to pathogens, or where limited data were available, homology with other antigens. The final multipathogen protein microarray included 1,607 proteins from 30 different pathogens (Table 1).

**Figure 2:**
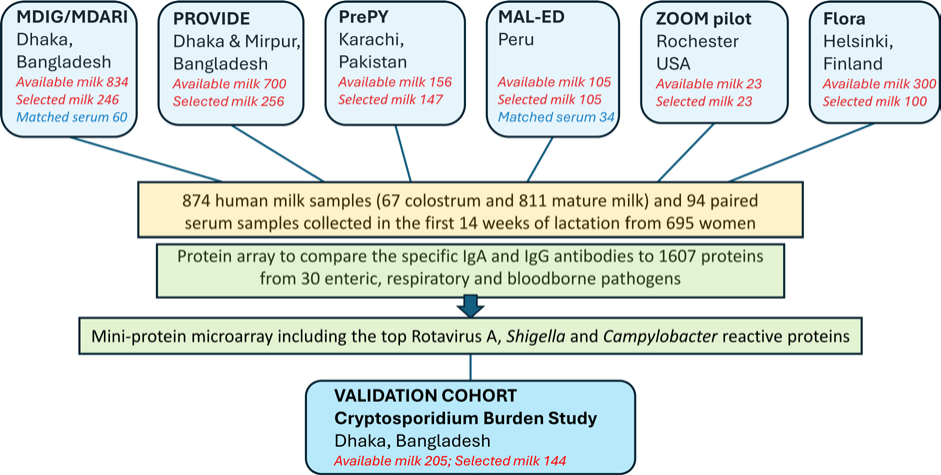
Flowchart of samples utilized in the study. Human milk samples from 6 cohorts in 5 countries were available for assessment of antibody profiles utilizing a protein microarray. As a validation cohort, we sourced an independent set of samples from a mother-infant paired birth cohort from Bangladesh (Cryptosporidium Burden Study) to probe against a mini protein microarray. All the samples available from the smaller studies were assayed at desired time points, and for larger cohorts (200 or more samples), approximately one-third of the samples were selected as representative of the whole cohort or based on the case-control design to include positive and negative infant infectious outcomes where available.

In total, 878 human milk samples (67 [7%] colostrum and 811 [93%] mature milk), which were collected from 695 women in Finland, U.S., Pakistan, Peru, and Bangladesh were assessed (Figure 2 and Supplemental Table 1; supplemental material available online with this article; https://doi.org/10.1172/JCI168789DS1). In addition, 94 matched maternal serum samples came from 60 12-week postpartum Bangladeshi mothers and 34 6-week postpartum Peruvian mothers.

### Profiles of human milk IgA and IgG antigen binding differ by economic and geographic region.

**Figure 3:**
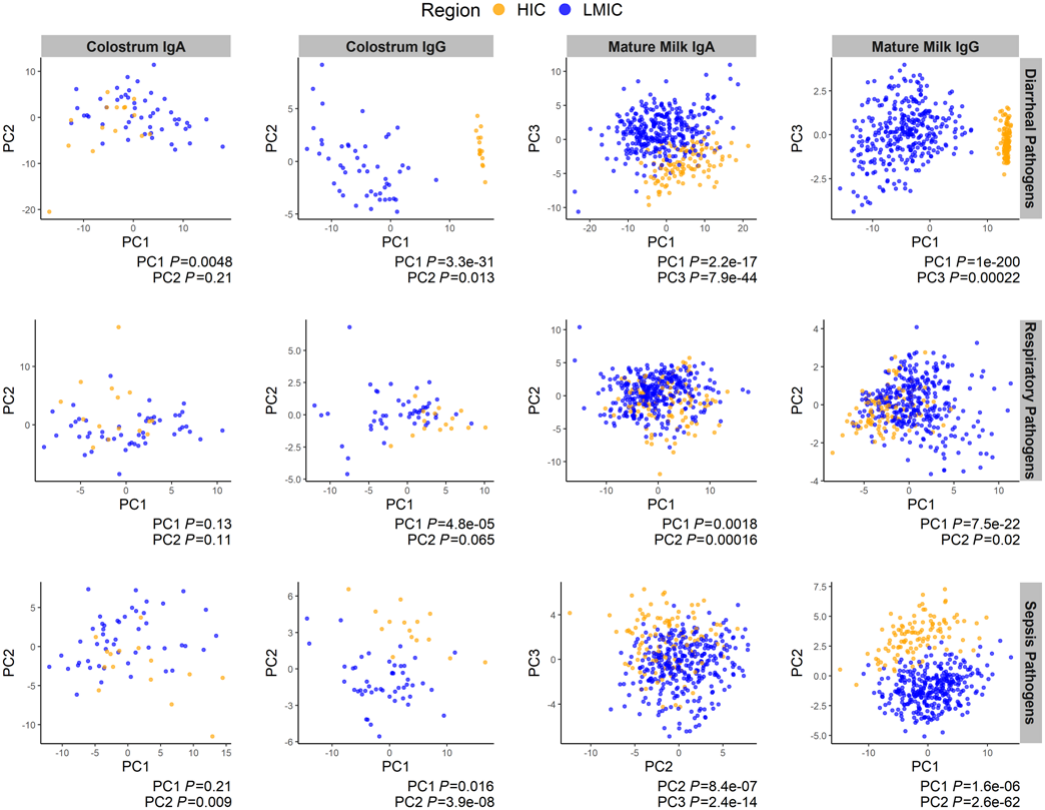
Principal component analysis by economic classification for colostrum and mature human milk. The scatter plots show principal component (PC) values for each individual’s IgA and IgG responses as points colored by economic region. The top row of plots show responses against enteric pathogen antigens, the second row for respiratory pathogen antigens, and the third row for sepsis-related pathogen antigens. The 2 leftmost columns of plots show colostrum IgA and IgG responses, and the 2 rightmost columns show mature milk IgA and IgG responses. Samples from HICs are shown in orange points, and samples from low- and middle-income countries LMICs are shown in blue points. Colostrum was available from Finland (*n* = 15) and Pakistan (*n* = 49); 1 mature milk sample per mother was available from Finland (*n* = 85), Rochester, New York, U.S. (*n* = 23), Peru (*n* = 34), Bangladesh (*n* = 246), and Pakistan (*n* = 49). *t* test *P* values for PC comparisons between HIC and LMIC are shown in captions below each plot. The 2 PCs with the lowest *P* values (PC1, PC2 or PC3) for each comparison were plotted.

First, to assess the overall differences between economic regions in IgA and IgG antibody repertoires, principal component analysis was performed separately on colostrum samples and mature milk samples for both IgA and IgG, and the responses to enteric, respiratory, and bloodborne pathogen proteins were analyzed independently (Figure 3). IgA responses in colostrum and mature milk between LMIC and HIC populations were most clearly delineated for enteric pathogens. Mature milk IgA responses against respiratory and sepsis-related pathogens were also significantly different between LMICs and HICs. IgG responses against all 3 types of pathogens clustered tightly by economic classification for both colostrum and mature milk. PCA results by individual countries are shown in Supplemental Figure 1. These data further indicate differences in antibody profiles within the HIC and LMIC cohorts.

**Figure 4:**
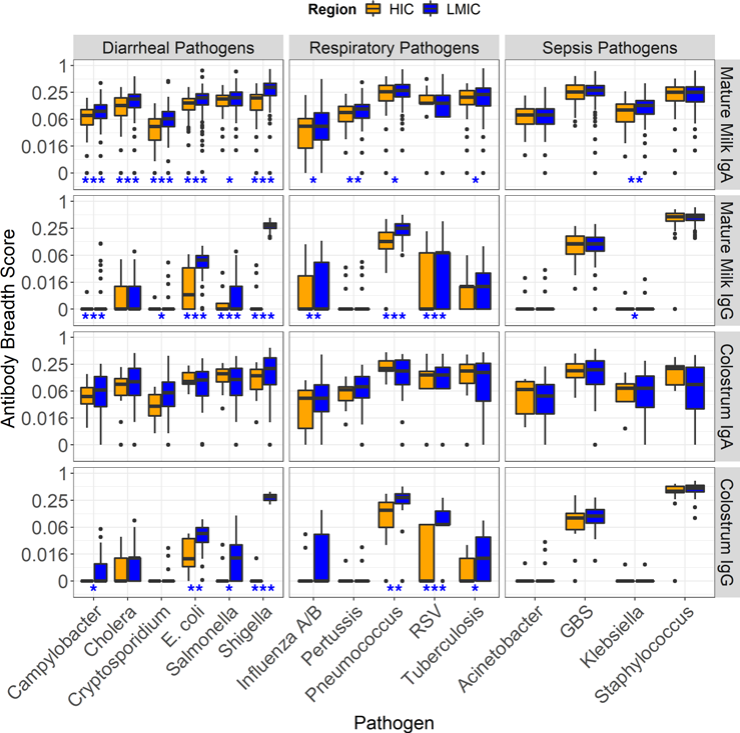
Pathogen-specific IgA and IgG breadth scores in mature milk and colostrum by economic classification. The box plots show comparisons of mature milk and colostrum IgA and IgG breadth scores (row headers), defined as the proportion of seropositive (normalized signal ≥ 1.0) antigens per pathogen for each individual (e.g., 20 of 80 positive responses = 0.25 breadth score). The column headers indicate the type of pathogens displayed in each column: enteric, respiratory, and sepsis-related pathogens. Rotavirus and Adenovirus 40/41 were omitted from the enteric pathogens column due to low numbers of reactive antigens. The x axes show each pathogen grouped by HIC (orange boxes) and LMIC (blue boxes) classifications. The y-axes show the IgA or IgG breadth scores on a logarithmic scale with the boxes representing the median and interquartile range. Significant differences by Wilcoxon’s rank sum tests are shown by blue asterisks below each pathogen: *P ≤ 0.05, ** *P* ≤ 0.005, ** P* ≤ 0.0005. Mature milk samples (*n* = 438) were included from the latest sample collection for each cohort and were at least 6 weeks postpartum. Colostrum samples (*n* = 64) were collected in the first 5 days. *E*. *coli*, diarrheagenic types EAEC, EPEC and ETEC; RSV, respiratory syncytial virus; GBS, group B *Streptococcus*.

To understand the potential difference in per-pathogen antigenic coverage between HIC and LMIC, an IgA and IgG antibody “breadth score” for each of the enteric, respiratory, and sepsis pathogens were assessed. Breadth score was calculated as the sum of seropositive responses (normalized signal intensity ≥ 1.0) per pathogen divided by the total number of probes for the corresponding pathogen, i.e., the proportion of positive probes. Comparisons of the distributions of antibody breadth scores between LMIC and HIC populations for enteric, respiratory, and sepsis-related pathogens are summarized in Figure 4. Mature milk IgA and IgG from mothers in LMIC was reactive to a higher number of enteric antigens (except for cholera) than that from HICs (Figure 4). Whereas IgG breadth scores were higher for several enteric pathogens in LMICs than HICs, colostrum IgA breadth scores were comparable. Further comparisons of IgA breadth scores between countries showed that women in Finland did not significantly differ from those in the U.S., but were most different from those in Bangladesh, followed by Pakistan and then Peru, which were all similar (Supplemental Figure 2). Pair-wise comparison of mature milk IgG between geographic regions showed findings similar to IgA, with *Shigella* being the most notable pathogen with a higher breadth score in the LMIC than HIC populations (Supplemental Figure 3).

Among the respiratory pathogens, LMICs had significantly higher mature milk IgA breadth scores for influenza A/B, *Bordetella pertussis*, pneumococcus, and *Mtb*, while the IgG breadth scores in the mature milk or colostrum were higher for influenza A/B, and particularly for pneumococcus and RSV (Figure 4). Pair-wise comparisons between countries did not show differences for mature milk IgA breadth scores (Supplemental Figure 4), but women from Bangladesh and Pakistan both had higher IgG breadth scores for pneumococcus and RSV than Finland and the U.S. (Supplemental Figure 5). Peru had pneumococcal breadth scores similar to the HICs.

There were few notable differential breadth scores among the sepsis-related pathogens (Figure 4). Mature milk from women from LMICs had higher IgA and IgG breadth scores to *Klebsiella pneumoniae* proteins, although IgG breadth scores were extremely low for both LMICs and HICs. Sepsis pathogen breadth scores were comparable between countries (Supplemental Figures 6 and 7). All statistical results are available in Supplemental Tables 1–3.

In summary, these data show that, compared with HIC, IgA and IgG in milk of women in LMICs are reactive to a higher number of antigens per pathogen for all enteric pathogens tested. LMIC women’s milk antibodies are also more reactive to a higher number of antigens from respiratory pathogens, but the reactive isotype differs between pathogens with IgG being reactive to more numerous RSV and pneumococcus antigens. HIC mothers do not have antibodies that are reactive to a higher number of antigens from any pathogens.

### Antibody responses in human milk are distinct from maternal serum responses.

**Figure 5:**
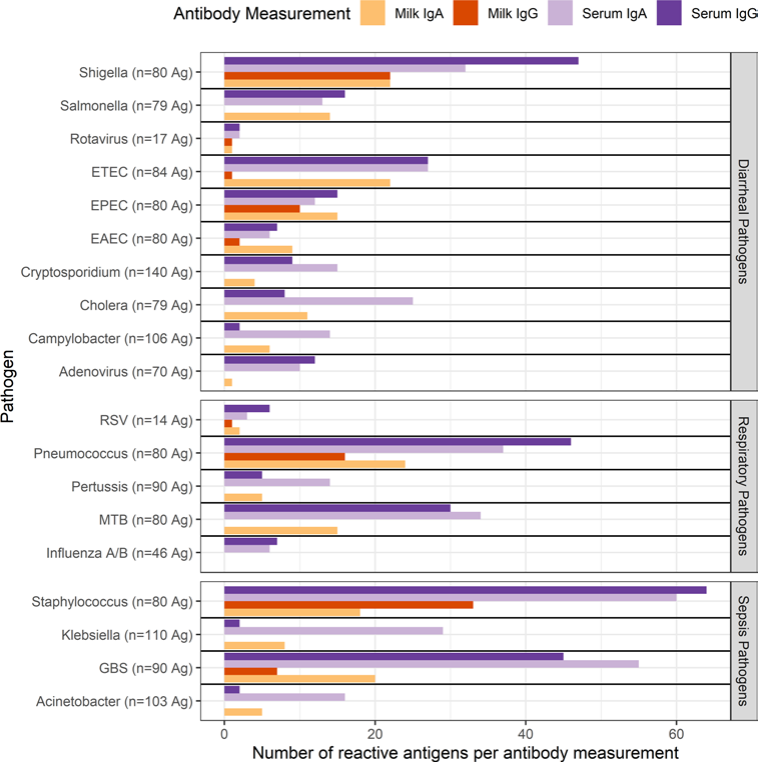
Comparison of antigen-specific recognition of IgA and IgG for enteric, respiratory, and sepsis pathogens in human milk and serum. The horizontal bar plots show the number of antigens bound by IgA for each pathogen by sample type and antibody isotype. Pathogens are shown on the y axis, grouped by disease category, with the total number of antigens (“Ag”) present on the multipathogen protein microarray in parentheses. The x axis shows the number of antigens from each pathogen that were reactive. Reactive antigens were defined as antigens with median IgA concentrations of at least 1.0 in normalized signal intensity. Only samples from the Peru and Bangladesh (MDIG) cohorts, which had paired serum and human milk samples at 12 weeks or later postpartum (*n* = 93 participants; 1 of the 94 participants with paired serum and milk samples did not have a later mature milk sample), were included in this analysis. Note that the total number of antigens differs between the pathogens and therefore the number of reactive antigens does not reflect relative reactivities between the pathogens.

To compare the mucosal and the systemic antibody responses across enteric, respiratory, and sepsis pathogens, paired milk and serum samples available from Peru and Bangladesh cohorts were assessed for IgA and IgG responses (Figure 2 and Supplemental Table 1). We first calculated the total number of reactive antigens of IgA and IgG for each specimen type (human milk or serum) (Figure 5). In general, *Shigella*, EPEC, pneumococcus, and *Staphylococcus* elicited both IgA and IgG responses in serum and milk; typically, serum antibodies were reactive to more antigens than those in milk and more reactive for IgG than for IgA. However, *Cryptosporidium*, *Campylobacter jejuni, Klebsiella pneumoniae,* GBS, and *Acinetobacter baumannii* had more antigens reactive to serum IgA than IgG responses, and among those, *Campylobacter jejuni*, *Klebsiella pneumoniae,* and *Acinetobacter baumannii* elicited a predominant IgA response in both milk and serum. Milk IgG responses were most rare and were not elicited at all for several pathogens, including *Salmonella*, *Cryptosporidium*, *Campylobacter jejuni,* adenovirus, pertussis, *Mtb, Klebsiella pneumoniae*, and *Acinetobacter baumannii*, or they were relatively poor, as in the case of ETEC and EAEC. There were no pathogens that generated both an IgA and IgG antibody response in human milk but not in serum. The viruses, adenovirus 40 and 41, influenza A and B, rotavirus A and RSV, elicited predominantly serum IgG and IgA responses.

Additionally, the overlap in reactivity for each specific antigen across specimen types and isotypes was tallied and visualized using “upset” plots (Supplemental Figure 8 and Supplemental Table 4) (31). *Shigella* spp., EPEC, pneumococcus, *Staphylococcus*, and GBS had the most numerous IgA- and IgG-reactive antigens shared between serum and milk. There were no pathogens where an antigen would have elicited all but serum IgG responses or all but serum IgA responses. While there are several pathogens where a serum-only response without milk response was seen, only EAEC and *Salmonella* had some antigens that reacted only with milk IgA and no pathogens where only a milk IgG response was seen. *Cryptosporidium* spp*.*, *Campylobacter jejuni*, *Klebsiella pneumoniae,* and *Acinetobacter baumannii* had a predominant IgA response in both milk and serum, with most milk IgA-reactive antigens also being reactive to serum IgA, but with some antigens uniquely recognized by serum IgA. Adenovirus and influenza tended to have antigens uniquely reactive to serum IgG or serum IgA.

In summary, these data suggest differences between pathogens in eliciting IgA versus IgG antibody responses, which are likely in part due to the invasiveness of the pathogen, with *Shigella*, EPEC, pneumococcus and *Staphylococcus* as examples of those with broad antibody responses across both isotypes in milk and serum, whereas *Cryptosporidium* and *Campylobacter* elicited predominantly an IgA response.

### The magnitude of human milk IgA and IgG responses is regulated by the duration of lactation, economic and geographic region, total immunoglobulin concentrations, BMI, and parity.

**Figure 6:**
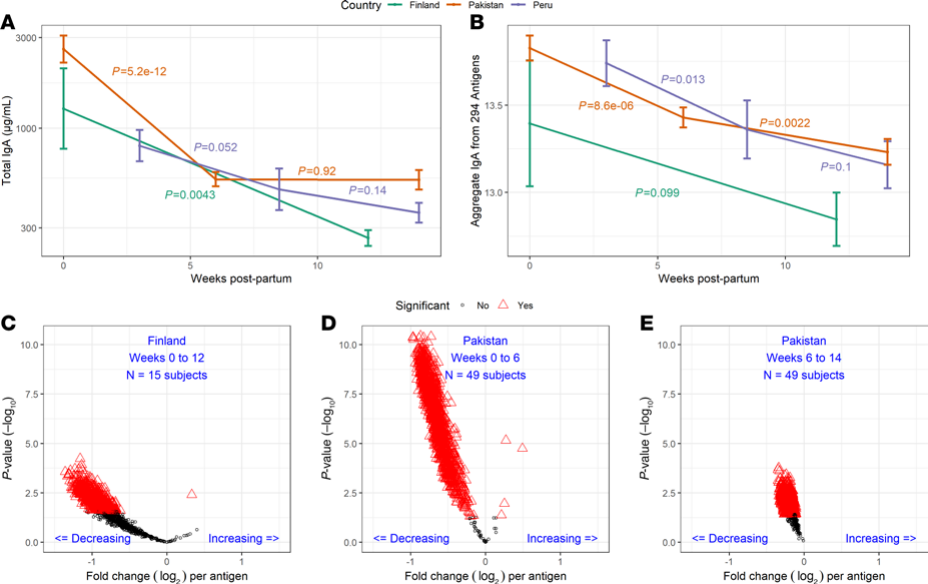
Total IgA and pathogen-specific IgA concentrations decline from colostrum to mature human milk. (**A** and **B**) The line plots show (**A**) μg/mL of total IgA in human milk and (**B**) the mean Log_2_ signal intensity of IgA antibodies specific for 294 reactive pathogen antigens on the multipathogen protein microarray over 12 to 14 weeks postpartum in Finland (*n* = 15 subjects), Pakistan (*n* = 49 subjects), and Peru (*n* = 9 subjects). The vertical bars represent the SEM. Paired *t* test *P* values are shown between time points and colored according to cohort. (**C**–**E**) The volcano plots show the difference between pathogen-specific IgA concentrations between time points for (**C**) Finland and (**D** and **E**) Pakistan. Comparison of samples from Peru is not shown due to low number of week 0 colostrum samples (*n* = 3). Each marker represents an antigen on the multipathogen protein microarray; red open triangles represent IgA responses to individual antigens that are significant after correction for the FDR and black open circles represent IgA responses to individual antigens that were not statistically significant. The x axes show mean differences between time points, and the y axes show the inverse Log_10_ *P* value from paired *t* tests.

To assess the factors associated with antibody concentrations, we first analyzed the effect of duration of lactation utilizing longitudinal human milk samples available from the Finland, Pakistan, and Peru cohorts (Figure 2 and Supplemental Table 1). Total and specific IgA antibody concentrations decreased significantly over the first 12-to-14 weeks of lactation in each cohort (Figure 6, A and B). Utilizing samples from Pakistan, we were able to establish that there was a significant decline in the first 6 weeks but not weeks 6 to 14 for both total IgA (*P* < 1 × 10^–11^ versus *P* = 0.9) and a significant decrease in aggregate specific IgA for 284 antigens (*P* < 1 × 10^–5^ versus *P* = 0.002). The comparison of concentrations of specific antibody responses between time points is further illustrated in Figure 6, C–E. Compared to IgA, total and antigen-specific concentrations of IgG were lower throughout lactation, and there were fewer shifts in IgG concentrations (Supplemental Figure 9). In samples from women in Pakistan, there was only a small, although significant, decrease in the concentrations of both total and specific IgG from 0 to 6 weeks (*P* = 0.003 and *P* = 0.02, respectively). In Finland and Peru, there were no significant changes in total IgG or specific IgG concentrations.

**Figure 7:**
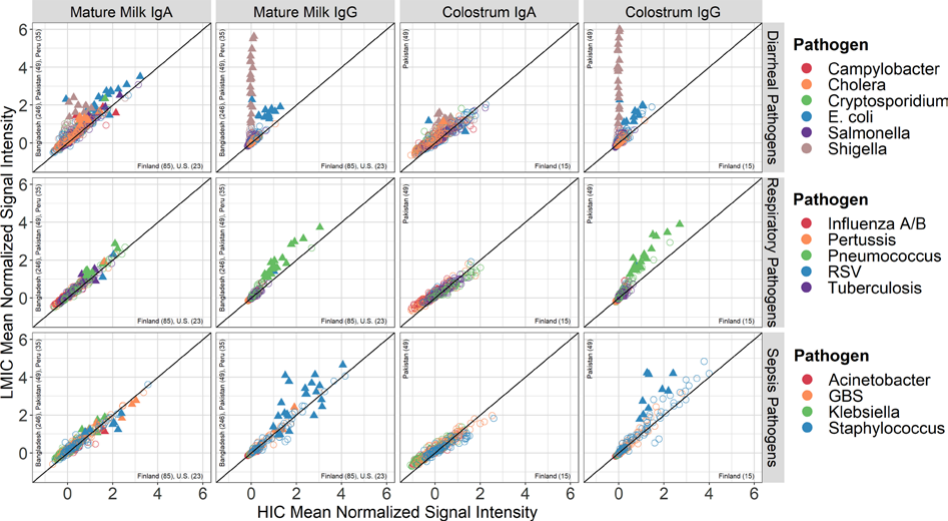
Pathogen-specific IgA and IgG concentrations in mature milk and colostrum by economic region. Scatter plots show IgA or IgG concentrations for each of the reactive antigens from enteric, respiratory, and sepsis-related pathogens (row headers) in mature milk and colostrum (column headers). Antigens classified as “reactive” were those having a median value ≥ 1.0 across the entire study population. Each point represents the mean normalized signal intensity for an individual antigen, colored by pathogen (row legends); solid triangles represent antigens with significant differential reactivity between cohorts by *t* tests after correction for the FDR. Y axes show means for samples from LMICs, and x axes show means for samples from HICs. The countries included in each plot are listed along the y-axis (LMICs) and x-axis (HICs) with sample sizes in parentheses. The solid diagonal line represents the line of identity (i.e., similar mean signal intensity between LMICs and HICs). Mature milk samples were those from the latest sample collection for each cohort and were at least 6 weeks postpartum. Colostrum samples were collected at 0 weeks. *E*. *coli*, diarrheagenic types EAEC, EPEC and ETEC; RSV, respiratory syncytial virus; GBS, group B *Streptococcus*.

To assess the effect of economic and geographic regions on the magnitude of antibody responses we performed group-wise comparisons of the normalized signal intensities of antibody binding to each protein (Figure 7). LMIC cohorts had higher IgA and IgG antibody concentrations in both mature milk and colostrum most notably for IgA and IgG to *Shigella* and diarrheagenic *E*. *coli*, and IgG to pneumococcal and staphylococcal proteins compared with HIC cohorts. In turn, HIC populations had higher responses in mature milk to a few individual antigens from *Staphylococcus*.

Pairwise comparisons between countries for enteric pathogen proteins further demonstrate the similarities and differences within and between HIC and LMIC populations with key differences driven by *Shigella* for IgA and IgG, and *E*. *coli*, *Cryptosporidium,* and cholera for IgA (Supplemental Figures 2 and 3). For the respiratory pathogens, the differences were largely driven by pneumococcal, *Klebsiella*, GBS, and *Mtb* antigens for IgA and pneumococcal antigens for IgG (Supplemental Figures 4 and 5). Regarding sepsis pathogens, HICs and LMICs differed for staphylococcal IgG antibody concentrations (Supplemental Figures 6 and 7).

Lastly, we utilized mature human milk samples to understand the effect of maternal factors such as parity, nutritional status, age, and education on IgA and IgG antibody concentrations against all pathogen proteins on the multipathogen protein microarray using multivariable linear mixed effects regression and ordinary least squares (OLS) regression (Supplemental Figures 10 and 11). We used mature milk samples because the concentrations stabilized after 6 weeks of lactation (Figure 5). Besides economic region, human milk total IgA and BMI were negatively associated with aggregate IgA antibody concentrations (i.e., mean of reactive antigens), although the effect size for human milk total IgA was small (Table 2). For IgG antibodies, parity was negatively associated and total IgG positively associated with the IgG antibody concentrations (Supplemental Table 2). Data on maternal age and highest level of education were only available in LMICs and showed no associations with antibody concentrations. In summary, besides the differences by economic regions and decrease over the duration of lactation, human milk antibody concentrations were lower in mothers with high BMI and parity.

### Human milk IgA antibodies are associated with protection against rotavirus but greater risk of Campylobacter infection.

**Figure 8:**
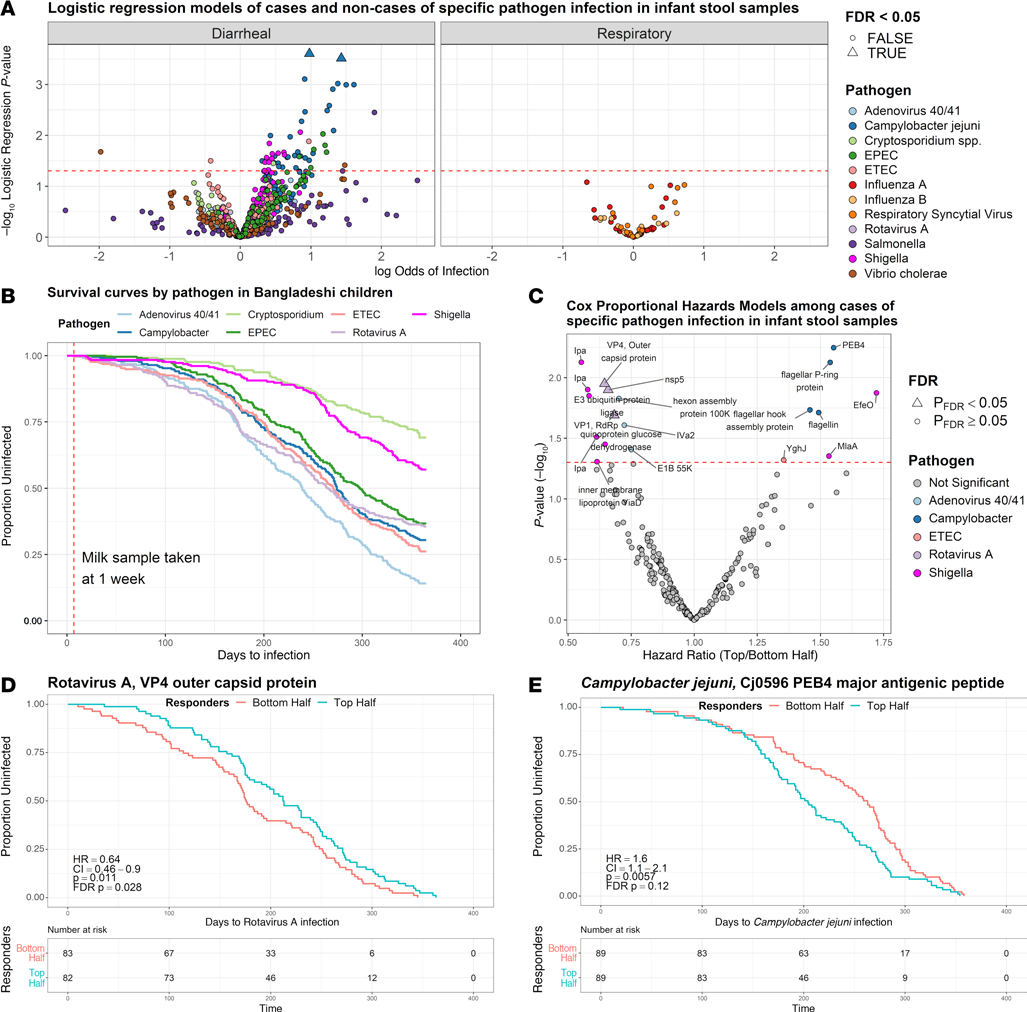
Association of human milk IgA with infection in breastfed infants. (**A**) Association of IgA binding to each pathogen for infants subsequently infected with the specific pathogen or not. The log odds from logistic regression of enteric (left) or respiratory (right) infection with increasing IgA binding (x axis) in infants during 1 year and 6 months of follow up, respectively, are shown with the inverse log_10_ *P* value (y axis). Associations significant after correction for the FDR are shown in colored triangles. Samples analyzed for diarrheal illness were from the Bangladesh PROVIDE cohort (*n* = 256) and for respiratory illness were from the Bangladesh MDIG cohort (*n* = 246). (**B**) Survival curves of 256 infants from the PROVIDE cohort for enteric pathogens detected by PCR. (**C**) Hazard ratios of infants during the first year of life divided into the top and bottom halves of mothers’ milk IgA responses for each antigen that was reactive in at least 10% of PROVIDE cohort women. Milk samples included were from mothers with infants that subsequently had pathogen-specific infection. Values below 1.0 represent lower risk of infection in the top half of milk IgA responses compared with the bottom half. For unadjusted *P* values less than 0.05, antigens were colored (otherwise grey), FDR-adjusted *P* values less than 0.05 were plotted as triangles. (**D** and **E**) Representative Rotavirus A antigen (**D**) and *Campylobacter jejuni* antigen (**E**) corresponding to the samples included in the models shown in the volcano plot (**C**). The risk tables show the number at risk during 100-day intervals. The Rotavirus A VP4 outer capsid protein is representative of antibodies associated with longer time to infection, while the *C*. *jejuni* PEB4 major antigenic peptide (Cj0596) represents antibodies associated with a shorter time to infection. HR, Cox model coefficient for the hazard ratio; CI, confidence interval; *P*, log-rank test *P* value; FDR *P*, adjusted *P* value.

Infection and disease outcomes in breastfed infants were available for the 2 Bangladesh cohorts. For the PROVIDE cohort, human milk samples collected at approximately 1 week postpartum from women whose infants had at least 1 episode of enteric illness were analyzed for association of antibodies with enteric pathogen outcomes during the first year of life. In the MDIG cohort, human milk collected at approximately 13 weeks postpartum from women whose infants had at least 1 nasal swab collected after the human milk sample was taken, as well as evidence of partial, predominant, or exclusive breastfeeding at least until the time of the index swab collection, were analyzed for association with subsequent influenza and RSV infections through the first 6 months of life. Importantly, samples from the MDIG study were selected and stratified into 2 equally sized groups on the basis of whether the infant had a nasal swab collected that tested positive for RSV and/or influenza A and/or influenza B after collection of the human milk sample. Among infants in the cohort with one or more microbiologically confirmed nasal swab for RSV and/or influenza A/B, mother-infant pairs were preferentially selected for this case-control study if the swab tested positive for RSV and if the cases/episodes had a more severe clinical presentation (i.e., lower respiratory tract infection). Controls were mother-infant pairs where the infant had any type of clinical acute respiratory infection for which the nasal swab tested negative for RSV and influenza A/B after collection of the human milk sample. Pathogens in both the PROVIDE and MDIG studies were detected by PCR-based assays. Additionally, in the PROVIDE cohort, infections were classified as causative of disease or not. Analysis of IgA response on the odds of corresponding pathogen infection by logistic regression showed few significant associations among diarrheal and respiratory pathogens (Figure 8A), although a general trend was observed for higher antibodies in human milk associated with infants that had subsequent enteric infections. Because there was a long period of follow up between early human milk sampling and illness outcome in the PROVIDE samples (up to the second half of the first year of life for some) (Figure 8B) and because it was not possible to distinguish infants without infection as those resistant to the pathogen or those that remained unexposed, we performed an exploratory analysis to assess if there were associations between human milk antibodies and time until infection with enteric pathogens or attributable illness. This approach has been useful in other disease models with heterogeneous exposure in the population (32). We used multivariable Cox proportional hazards (CPH) models to explore associations of antibodies and the hazard function, correcting for sex, days of exclusive breastfeeding, the WHO child growth standard length-for-age z-score (LAZ) at enrollment, parity, maternal age, maternal BMI, maternal education in years, household income/expenditures, and ordinal category of improved drinking water treatment. For each antigen, IgA and IgG responses were categorized by the median as top or bottom-half responders (33). Among those antigens for which positive IgA responses were seen in at least 10% of women, there was a trending association between higher human milk IgA responses against rotavirus, adenovirus 40/41, and *Shigella* and a lower incidence of specific infection in the infant, i.e., the most significant associations had hazard ratios below 1, indicating a protective effect (Figure 8C). After correction for the FDR, the correlation for 3 Rotavirus A antigens remained significant: the top half of IgA responders to the VP4 outer capsid protein, nonstructural protein 5, and VP1 RNA-directed RNA polymerase (RdRp) had infants with a significant delay in time to infection. An example Kaplan-Meier plot for Rotavirus A VP4 is shown in Figure 8D. IgA responses to these Rotavirus A proteins, adenovirus 40/41, and *Shigella antigens* were not significantly associated with a delay to diarrheal disease caused by these pathogens in the infant (Supplemental Figure 12, A and B), with the exception of Rotavirus A VP4, which was similarly associated with reduced time to Rotavirus A–attributable diarrhea before correction for the FDR (Supplemental Figure 12C). In contrast, the top half of *Campylobacter* IgA responders had a child with a higher risk of infection and diarrheal disease caused by *Campylobacter*, and similar trends were observed for EPEC infection and ETEC-attributable diarrhea; however, these were not significant after correction for the FDR (Figure 8C and Supplemental Figure 12). An example Kaplan-Meier plot for specific IgA to *Campylobacter jejuni* Cj0596 major antigenic peptide PEB-cell binding factor, also known as PEB4, is shown in Figure 8E for PCR-confirmed infection and Supplemental Figure 12D for attributable disease. Inclusion of all antigens in the models, even those for which less than 10% of women had an IgA response, showed a similar finding that higher human milk IgA concentrations against Rotavirus A proteins were significantly associated with a delayed time to infection in the infant, and higher IgA concentrations against *Campylobacter* proteins were significantly associated with a reduced time to infection, even after correction for the FDR (Supplemental Figure 13). For comparison, we performed CPH models on all the 256 human milk samples irrespective of whether infants had confirmed exposures or not, and, like the logistic regression models in Figure 8A, the strongest associations for IgA antibody concentrations were with increased risk of infection and diarrheal illness (Supplemental Figure 14).

**Figure 9:**
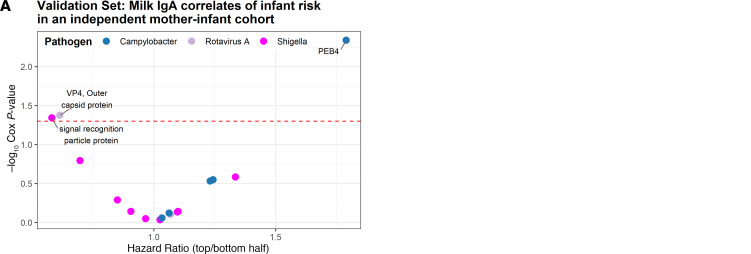
Validation of human milk IgA correlates of risk in an independent mother-infant birth cohort. (**A**) Hazard ratio of infants during the first year of life divided into the top and bottom halves of mothers’ milk IgA responses for antigens included in a validation mini-protein microarray. Milk samples were from women in the independent Cryptosporidium Burden Study cohort (Validation set, *n* = 144 milk samples). Only proteins that were reactive in at least 10% of women in the Cryptosporidium Burden Study are shown. (**B**) Hazard ratio and 95% CIs from Cox proportional hazards models of infants during the first year of life divided into the top and bottom half of mothers’ milk IgA responders against *Campylobacter jejuni* PEB4 and Rotavirus A VP4. *P*,Cox regression *P* value; FDR, adjusted *P* value.

To assess generalizability of the finding that milk IgA responses were associated with delayed or shorter time to infection, we sourced an independent set of samples from a mother-infant paired birth cohort in the same region of Bangladesh (Cryptosporidium Burden Study, CBS (34)). These human milk samples were tested on a mini-protein microarray that included the Rotavirus A, *Shigella,* and *Campylobacter* proteins identified from the PROVIDE study as correlates of delay or reduction in time to infection or diarrheal illness (unadjusted *P* values < 0.05 in CPH models) (Figure 8C and Supplemental Figure 13). The concentration of IgA to Rotavirus A VP4 outer capsid protein was associated with a delayed time to infection in the infant (HR: 0.61, CI: 0.38–0.98, *P* = 0.042, *P*_FDR_ = 0.2, *n* = 92 cases,), and the concentration of IgA to *Campylobacter jejuni* PEB4 major antigenic protein was associated with a reduced time to infection in the child (HR: 1.79, CI: 1.2–2.7, *P* = 0.005, *P*_FDR_ = 0.08, *n* = 116 cases) (Figure 9).

## Discussion

Based on measuring the specific IgA and IgG to 1,607 proteins from 30 pathogens in 878 milk samples collected from 695 women across 5 countries (Finland, U.S., Pakistan, Peru, and Bangladesh), we showed significant differences in antibody concentrations and repertoires directed at enteric, respiratory, and bloodborne pathogens responsible for substantial morbidity and mortality between LMICs and HICs. We also identified maternal parity and BMI as individual-level factors that were associated with their concentrations. The antibody repertoires in the U.S. and Finland were most similar to one another and most different from Bangladeshi cohorts. Whereas some pathogens commonly elicited both IgA and IgG antibodies shared between human milk and serum, others induced a dominant IgA response in milk and serum, or a predominantly serum IgG and IgA response (i.e., viruses) depending on the route of infection and other characteristics. Finally, we showed that higher milk-specific IgA was associated with delayed time to rotavirus infection, which may indicate a role for human milk antibodies in protection against enteric diseases. Our work demonstrated important international variations in antipathogen antibody repertoires in human milk, whereas previous studies have focused on single cohorts or a single pathogen and have not included the comparison between serum and human milk antibody profiles or clinical metadata to analyze the effect of maternal factors on antibody concentrations and infectious outcomes.

Compared with our previous report also utilizing a similar high-throughput protein microarray (30), we expanded the panel of enteric pathogens and showed that a larger number of antigens were recognized by human milk IgA antibodies in LMICs than in HICs from *Campylobacter*, cholera, *Cryptosporidium*, diarrheagenic *E*. *coli*, *Salmonella,* and *Shigella*. Among the respiratory pathogens, IgA antibodies in mature milk from mothers from LMICs recognized a larger number of antigens from influenza A/B, *Bordetella pertussis*, *Streptococcus pneumoniae,* and *Mtb*. The IgA responses are considered to reflect prior maternal infection or exposure at mucosal surfaces, because human milk IgA is produced by the mammary gland plasma cells that have migrated from the mother’s gut or respiratory mucosa via the “enteromammary link”. The IgA produced in the lamina propria is transported across epithelial cells by the polymeric immunoglobulin receptor (pIgR) resulting in SIgA. Although our assay measured IgA and not specifically SIgA, most of the IgA detected in human milk is SIgA. In contrast to IgA, the source of IgG in human milk is either specific IgG-secreting cells found in human milk, which have a mucosal homing profile (35) or plasma-derived IgG (36). The specificity of human milk IgG may therefore be reflective of how much specific IgG is produced by mucosal versus peripheral blood IgG-secreting cells, which may vary between mothers (37).

In addition to the antibody repertoire, the magnitude of antibody response to enteric and respiratory pathogens was higher in LMICs compared with HICs. Although the antibody concentrations are likely boosted with recent exposure (38), as IgA responses to microbes are constantly modified to reflect the microbiota present in the gut lumen (39), the memory of plasma cells homing to mammary glands during pregnancy potentially lasts for several years (40, 41). It has been well established that total immunoglobulin and at least some specific antibody concentrations decrease during lactation (42), which we here confirmed. We further found that among maternal factors, BMI was negatively related to the IgA and parity to the IgG antibody concentrations in human milk, the latter confirming previous findings that suggested this to be due to decreasing mammary secretory capacity in countries with fluctuations with food availability, disease outbreaks, and poor utilization of health care (43). Our novel findings may have societal implications, with increasing overweight and obesity rates associated with decreasing amounts of passive IgA antibodies provided to the offspring.

Some respiratory and enteric pathogens induced a dominant IgA response in milk and serum (*Campylobacter*, *Cryptosporidium*, pertussis*, Klebsiella,* and *Acinetobacter*), whereas others elicited both IgA and IgG antibodies with overlapping specificities in human milk and serum (*Shigella, EPEC, Streptococcus pneumoniae, Staphylococcus aureus,* and GBS*)*. These differences may reflect compartmentalization of the response based on the route and nature of exposure; localized exposure drives a mucosal IgA response, whereas invasive infection is more likely to lead to long-lasting plasma cells in bone marrow eliciting serum IgG responses. This led us to speculate that antibodies against some antigens arise from and protect against invasive disease, while other antigens recognized by human milk IgA arise from and protect against localized, mucosal exposure or disease. Among *Shigella flexneri* antigens, approximately half of the invasion plasmid antigens (Ipa) and proteins involved in the type III secretion system, including proteins from *mxi*-*spa* loci and Ipg proteins, were not reactive to human milk IgA but were reactive to serum IgA and IgG. However, the well-known Ipas, IpaA, IpaB, and IpaC, but not IpaD, as well as the enterotoxicity-related SepA protein elicited both serum and milk antibodies (44). The type of response may also affect the magnitude and type of protection provided to the infant, with mucosal IgA providing protection locally in the infants’ gut and upper respiratory tract whereas maternal systemic antibodies are transferred to an infant prenatally.

The disconnect between IgG and IgA transfer into milk is striking, particularly for *Cryptosporidium* and *Shigella,* and differs between LMIC and HIC. *Shigella* induces great IgG and IgA responses in milk in LMIC but only IgA responses in HIC. This may indicate more invasive infections in LMIC resulting in IgG responses, whereas IgA responses, which are potentially induced at mucosal tissues, were equally distributed between LMIC and HIC. Conversely, *Cryptosporidium* induces enhanced IgA responses in LMIC compared with HIC, but poor IgG responses in both LMIC and HIC, which may indicate increased frequency of *Cryptosporidium* exposure in LMIC limited to the mucosa resulting in an IgA-dominant response. Interestingly, both IgA and IgG responses to both pathogens are found in serum. Head-to-head comparison shows that *Shigella* induces both broadly universal responses across milk and serum IgA and IgG whereas *Cryptosporidium* responses are more serum limited.

sIgA is thought to be a first-line defense against foreign antigens and to inhibit inappropriate immune activation by microorganisms and antigens in the lumen of the intestinal and respiratory tracts through “immune exclusion” (45). Consistently, higher colostrum and mature milk antibody concentrations to enteric pathogens have been associated with protection against enteric diseases caused by *Shigella* (20), *Giardia lamblia* (21, 22), *Entamoeba histolytica,* and *Cryptosporidium* (23), *Campylobacter* (24),and *H*. *pylori* (46). In addition, IgA in colostrum has been shown to react to EPEC antigens in the feces of a breastfed infant (47), which may be responsible for the protective effect of breastfeeding on diarrheagenic *E*. *coli* strains (48). The concentration of anti-*Shigella* IgA specific to virulence plasmid–associated antigen in human milk was 8-fold higher in infants with asymptomatic *Shigella* (detected in stool) compared with those living in the same region but developing *Shigella*-attributed diarrhea (20). Similar findings were reported for *Giardia lamblia*-specific IgA in human milk (21). More recently, human milk–specific IgA was associated with time to acquire infection in the breastfed infant. Infants of mothers in the top half for human milk concentrations of IgA specific to *E*. *histolytica* had a significantly higher probability of survival free of *E*. *histolytica* infection through the first year of life, and high anti-*Cryptosporidium* IgA levels improved probability of survival free of *Cryptosporidium* infection (23). Finally, lower concentrations of *H*. *pylori*–specific IgA antibodies in human milk were associated with infection during the first month of life compared with infection at 6 months of age (46).

Here, we report that,in Bangladeshi infants with the cause of diarrhea confirmed by PCR, higher milk-specific IgA was associated with delayed time to rotavirus infection; a similar trend was observed for adenovirus 40/41 and *Shigella*. In contrast, higher specific IgA was associated with greater, not lower, incidence of *Campylobacter* infection, with a similar trend for EPEC and *Cryptosporidium*, suggesting that milk antibodies may be markers of exposure, rather than protection, for some pathogens (49). These associations may be reasonable when considering the dynamics of transmission for each pathogen. For example, rotavirus is highly transmissible person-to-person, especially among infants and children, and early antibodies may provide protection from infection when exposure occurs (50). In contrast, *Campylobacter* infection spreads poorly person-to-person but easily through contaminated food and contact with domestic animals; antibodies may serve as a marker of exposure (and likelihood of eventually being infected), regardless of antibody protective effects. Alternatively, bacterial-specific IgA may enable stabilized intestinal colonization and confer resistance to invasion by exogenous competitors (51). At the same time, the role of IgG antibodies in human milk is less well known and no associations with protection were detected in our study. Another study showed that the median human milk IgG (but not IgA) antibody concentration was lower in mothers of infants with RSV before infant acute respiratory illness than in those without RSV acute respiratory illness, supporting a potential role for IgG, but not IgA, antibodies in protection against RSV (52). A recent paper also showed that during exposure to neopathogens, maternal natural IgG antibodies to commensal bacteria in human milk protected offspring against enteric illness such as ETEC (53).

It is interesting that the most significant IgA response associated with delay to infection was the Rotavirus A VP4 outer capsid protein. VP4 is a spike protein proteolytically cleaved into VP5* and VP8* subunits before the virus can be activated (54), the latter a subunit vaccine candidate (55–58) as well as targeted regions of VP4 (59, 60). Neutralizing antibodies have been identified for both VP4* and VP8* (61, 62), as well as resistance to challenge (63). This and similar trends observed for *Shigella* and adenovirus 40/41 antigens suggests that functional IgA passively transferred to infants may contribute to protection against these pathogens. To ensure that this was not a chance finding, we performed similar experiments on an independent set of milk samples from mothers living in the same region of Bangladesh that participated in a different cohort study, the Cryptosporidium Burden Study (34). Using a mini-protein chip containing the top hits from our initial analysis, VP4 again was the most significant antibody response associated with delayed infection, while the *Campylobacter* PEB4 antigen–specific antibody was again associated with reduced time to infection. These results lend confidence to the associations observed in the PROVIDE cohort and support further exploration of the role of antigen-specific milk IgA responses in protection or as potential markers of exposure or risk.

There are several limitations to the study, which include that the antigens assessed here are restricted to protein antigens, although other important antigens also include glycans and lipids, such as lipopolysaccharides. Our methods only measure the free IgA in human milk, but not that combined with bacteria (64). Because samples were a convenience sample, the collection methods were not standardized across cohorts; however, samples were stored in –80°C and only thawed one or two times. Although this could result in subtle differences in antibody concentrations, trends between HICs and LMICs were similar to our previous report (30), in which sample collection was standardized between sites. Our analyses relating to disease outcomes were subject to confounding and selection biases due to only subsets of samples assessed; however, modeling the full PROVIDE cohort showed similar trends to the case-control analysis. Further, this exploratory analysis suggested that the relationship between the antibody and disease outcome is complex and often masked by missing clinical and epidemiological information regarding exposures. A major limitation of a convenience sample is the availability of samples with an adequate design for immune correlates analysis. The ideal for immune correlates analysis may be a longitudinal clinical study focused on fewer pathogens with careful control of exposure and maternal infections, pathogen detection, and frequent sampling. The pathogens tested are also not equally prevalent even in endemic areas. For example, exposure to key respiratory and enteric pathogens is nearly universal for some pathogens (*Shigella* and *E*. *coli*), but not for others (*Mtb* or bloodborne pathogens), limiting our power to detect protection against rare pathogens. In the case of influenza A, antigens included on the multipathogen array were from strains A/Victoria/361/2011 and A/California/04/2009, which may have been antigenically distinct from circulating strains in 2015 (65) and may have contributed to the lack of antibody associations with infection observed in our study. Lastly, for the antibody responses studied here, it is unknown whether antibodies reflect specific exposures/infections or cross-reactivity with other pathogens, commensal microbiota, or pathobionts. For example, recent exposure is unlikely to explain human milk IgA reactivity observed against some pathogens such as *Mtb* and cholera, for which exposure in these populations is negligible or insufficient to explain the high concentrations of reactivity seen; however, cross-reactive nonpathogenic organisms are present in the environment, such as the ubiquitous nontuberculosis *Mycobacteria* species (NTMs) (66). Supporting the possibility that the antigens chosen for *Mtb* are not specific to this species is the fact that the gene products of Rv0583c, Rv2396, and Rv3333c (lipoprotein LPQN, PE-PGRS family protein PE_PGRS41, and hypothetical proline rich protein, respectively, the 3 most-reactive *Mtb*-associated antigens studied here) are encoded by both *Mtb* and *Mycobacterium bovis*. Importantly, the high reactivity to *Mtb*-related antigens is likely not due to maternal or infant immunization, as tuberculosis vaccination with bacillus of Calmette and Guerin (BCG) broadly used in Finland was never recommended in the U.S. A remarkable strength of the study is the high-throughput protein microarray platform that allows simultaneous assessment of antibody repertoires to a library comprised of hundreds of antigen targets. The present study was unique in its representation of different economic and geographic regions and the inclusion of related metadata on BMI, education, parity, and infant enteric and respiratory infectious disease outcomes. An important strength of this study was gaining access to samples from the Cryptosporidium Burden Study, an independent cohort of dyads from the same region as the PROVIDE study, which served as a validation set for generalizability of the exploratory analysis. This set was limited by a smaller sample size and fewer specific cases of Rotavirus A, *Shigella,* and *Campylobacter* than the PROVIDE cohort. Despite reduced statistical power, the consistency between the top Rotavirus A and *Campylobacter* IgA responses provides a measure of confidence in the results described in the PROVIDE cohort.

In conclusion, this study comprises the first-of-a-kind reference database on human milk and paired serum IgG and IgA antibody reactivity and their regulation to a representative set of key pathogens responsible for substantial morbidity and mortality across the world. Future studies designed to investigate the impact of milk antibodies are required to confirm whether milk antibodies play a role in protection against rotavirus and other infectious diseases in the infant or represent biomarkers of exposure. Such data are important in order to design strategies to boost maternal antibody concentrations for the benefit of infant health.

## Methods

### Sex as a biological variable.

This study only included human milk from lactating women.

### Study design.

This retrospective case-control study was conducted using samples from 6 cohort studies/clinical trials conducted in 5 countries. In brief, all the samples available from the smaller studies were assayed at desired time points, and for larger cohorts (200 or more samples), approximately one-third of the samples were selected as representative of the whole cohort. A total of 874 human milk samples collected in the first 14 weeks of lactation (67 colostrum and 811 mature milk) from 695 women in 5 HICs and LMICs (Bangladesh, Finland, Peru, Pakistan, and the United States) were assessed to compare the specific IgA and IgG antibodies to 1,607 proteins from 30 enteric, respiratory, and bloodborne pathogens. In addition, 94 temporally matched, paired maternal serum samples were assayed to examine associations between immune profiles in serum and human milk. Cohorts included: (a) Maternal Vitamin D for Infant Growth (MDIG) trial (67) and the Maternal Vitamin D for ARI in Infancy (MDARI) (68) in Dhaka, Bangladesh; (b) PROVIDE study (69, 70) to assess the impact of enteric inflammation on polio and rotavirus vaccine responses and infant health including diarrheal outcomes in Dhaka and Mirpur, Bangladesh; (c) PrePY study on the epidemiology of pertussis in infants in Karachi, Pakistan (71); (d) Peru cohort of the MAL-ED study, “Interactions of Enteric Infections and Malnutrition and the Consequences for Child Health and Development” (72); (e) urban/suburban arm of the Rochester, NY, USA ZOOM Lifestyle and allergic diseases pilot study (73, 74); and (f) nonsupplemented arm of the Flora randomized, double blind, placebo-controlled trial of pre and probiotic supplementation in prevention of allergic diseases in Helsinki area, Finland (75). A detailed description of the methods and results from each of the 6 studies have been published elsewhere (Supplemental Table 1), methods and choice of samples are summarized in the Supplemental Methods. Metadata available, including maternal age, BMI, parity, urban-versus-rural housing, and education was collected. In addition, milk samples from an independent cohort of mother-infant pairs were tested as a validation set for the associations observed in the PROVIDE cohort using Cox regression models. These samples came from the Cryptosporidium Burden Study (National Clinical Trial Identifier: NCT02764918) in Dhaka and Mirpur, Bangladesh and included 144 milk samples taken from a similar time point to the PROVIDE study at approximately 1 month postpartum.

### Processing of human milk.

Methods used to prepare the milk for protein array analysis were adapted from Järvinen et al. (76). Briefly, after being thawed on ice and homogenized, whole milk was centrifuged for 15 minutes at 15,000 *g* at 4°C. Avoiding both the fat layer and cell pellet, the skim layer was then carefully removed. This procedure was repeated on the skim fraction, and the resultant supernatant was refrozen at –80°C until it was analyzed for its immune specificity.

### Antibody profiling of human milk and maternal serum.

Multipathogen protein microarrays were designed, fabricated, and probed for IgA and IgG binding at Antigen Discovery Inc. (ADI). All samples were assayed in a single batch. Selection of antigens for protein arrays, fabrication, and quality control and probing details are included in the Supplemental Methods. In brief, milk samples were diluted to a 1:5 final concentration in a 1.5 mg/mL *E*. *coli* lysate solution (Antigen Discovery Inc.) in protein arraying buffer (Maine Manufacturing) and incubated at room temperature for 30 minutes before applying samples to arrays. Validation set milk samples from the Cryptosporidium Burden Study were diluted to a 1:15 final concentration, as per updated SOPs at the time of testing. Arrays were incubated overnight at 4°C with agitation, washed 3 times with TBS-0.05% Tween 20 (Thermo Fisher Scientific, diluted 20× in molecular grade water), and incubated with biotin-conjugated anti-Human IgA diluted 1:1,000 (Jackson ImmunoResearch; 109-065-011). Arrays were washed and incubated with streptavidin-conjugated SureLight P-3 (Columbia Biosciences) at room temperature, protected from light. Washed and dried microarray slides were scanned using a GenePix 4300A high-resolution microarray scanner (Molecular Devices) and signals were quantified using Mapix software (Innopsys). All further data processing was performed in R (http://www.R-project.org). Data were normalized by first transforming raw values using the base 2 logarithm, then subtracting the median signal intensity of the IVTT control spots for each sample. This procedure provides a relative measure of the specific antibody binding versus the nonspecific antibody binding to the IVTT expression system. With the normalized data, a value of 0.0 means that the intensity is no different than that of the IVTT controls, and a value of 1.0 indicates a doubling with respect to IVTT control spots.

### Statistics.

An a priori statistical analysis plan was drafted and reviewed before data acquisition and preprocessing, including primary and secondary objectives and samples size calculations, which is detailed in the Supplemental Methods. Assays and the initial set of analyses were performed blinded. Specific statistical analyses used are mentioned in each figure legend. Briefly, differences in signal intensity means and principal component (PC#) means between groups was assessed by *t* tests. Differences in means between time points of paired samples used paired *t* tests. Breadth scores were assessed using Wilcoxon’s rank sum test. Factors associated with antibody levels were tested using multivariable ordinary least squares (OLS) linear regression. The association of antibody levels with cases and noncases of pathogen infection or pathogen-attributable disease was assessed using logistic regression. Hazard ratios of infection or disease in participants grouped as higher or lower antibody responders was assessed using Cox proportional hazards regression and log-rank tests. All statistical test *P* values were adjusted for the FDR as detailed in the Supplemental Methods.

### Study approval.

Written informed consent was obtained from all participants. The study was approved by the Research Subjects Review Board of the University of Rochester Medical Center (#STUDY00000585) and by the local Ethics committees.

### Data and materials availability.

The data used in this study as well as the data values underlying manuscript figures are available in the Supporting Data Values file. Additional results from statistical analyses are available in Supplemental Tables 1–4.

## Author contributions

The following authors designed original studies and oversaw collection of samples: KMJ, RJL (Rochester, New York, USA); AKK, MK, ES (Flora study, Helsinki, Finland); PPY, MNK (MAL-ED, Loreto, Peru); NTI, SBO (Pakistan); DER, LGP, SKM, AAM (MDIG trial, Dhaka, Bangladesh); WAP, BDK, RH (PROVIDE study, Dhaka, Mirpur, Bangladesh); and WAP, RH, ASGF (Cryptosporidium Burden Study, Dhaka, Mirpur, Bangladesh). JJC, AES and KMJ conceptualized the study. JJC and AZR oversaw down selection of antigens for protein array analysis. AO and EH performed bioinformatics analysis for down selection of antigens for protein array analysis. JJC and AT designed protein arrays. JP and CH established clone library and expression of proteins. LBG and GR managed local biorepositories and databases. ADS and JT performed protein array assays. JM performed total IgA and IgG measurements. JJC was responsible for visualization of the data. JJC and KMJ were responsible for the funding acquisition. JJC and AZR performed bioinformatics and statistical analyses. KMJ was responsible for the project administration and supervision. JJC and KMJ wrote the original draft. JJC, AES, MK, SKM, LGP, BDK, RH, MNK, ES, SBO, DER, WAP, and KMJ reviewed and edited the final manuscript.

## Supplementary Material

Supplemental data

ICMJE disclosure forms

Supplemental tables 1-4

Supporting data values

## Figures and Tables

**Table 1:**
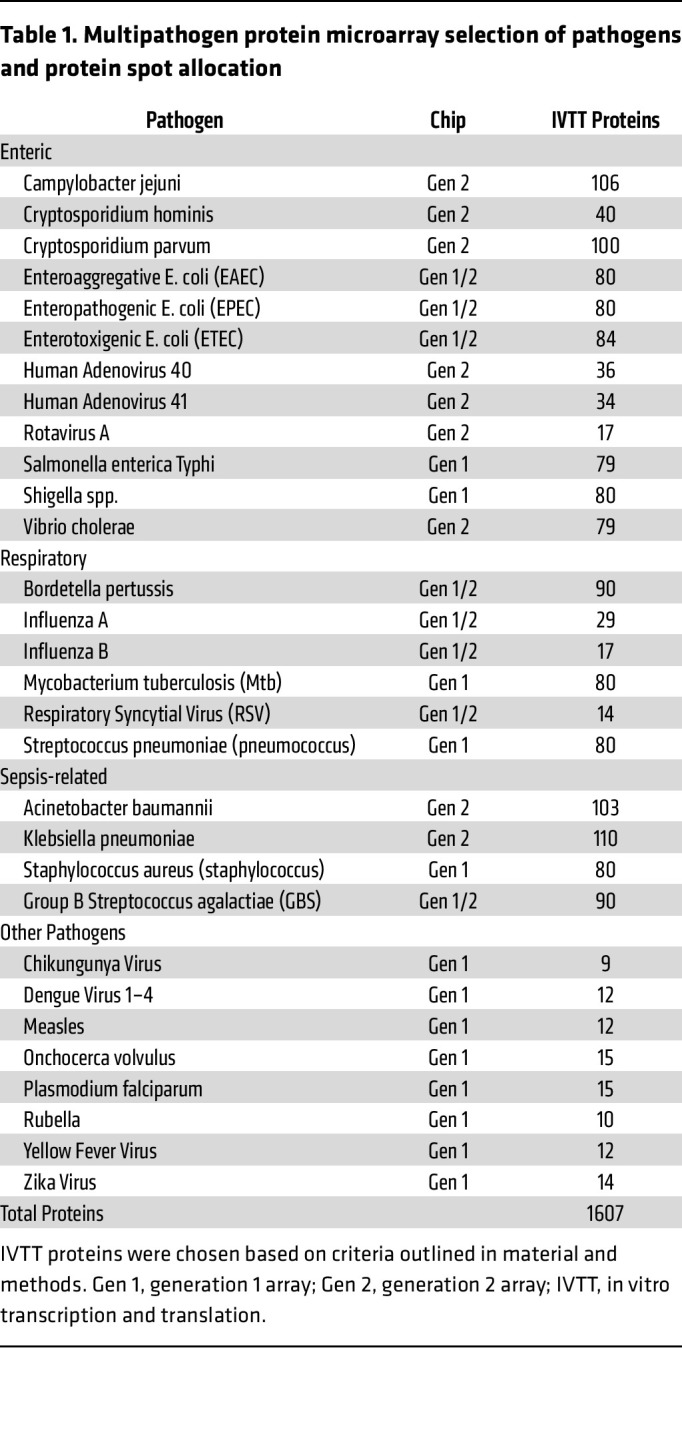
Multipathogen protein microarray selection of pathogens and protein spot allocation

**Table 2:**
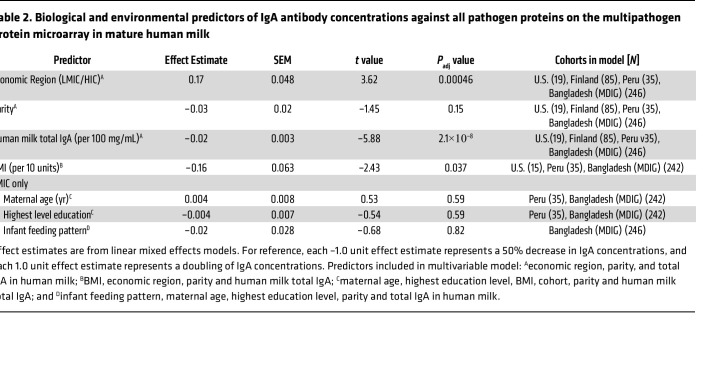
Biological and environmental predictors of IgA antibody concentrations against all pathogen proteins on the multipathogen protein microarray in mature human milk

## Version 1 08/01/2024

Electronic publication
