# Supplementary material for: Human milk antibodies to global pathogens reveal geographic and interindividual variations in IgA and IgG
Source: J Clin Invest. 2024 Jun 11;134(15):e168789. doi: 10.1172/JCI168789 (PMC11290967; doi:10.1172/JCI168789)
Supplement: Supplemental data [file jci-134-168789-s079.pdf]

SUPPLEMENTARY MATERIALS

Supplemental Methods

Study objectives. Study objectives were assigned prior to the study. They include Primary Objectives:

1. To determine the key enteric pathogens inducing colostrum and mature milk IgG and IgA antibody production and to characterize the differences in pathogen-specific antibody levels between developing and developed countries, and different geographic regions.

2. To assess a possible association between mother's milk antibody immunoprofile and disease outcomes of pathogen-confirmed diarrheal diseases (PROVIDE cohort) and respiratory episodes (MDIG/MDARI cohort), attributable to pathogen causes of diarrhea and respiratory illness in the children.

Secondary Objectives:

1. To compare whether antibody profiles are similar in colostrum and mature milk (and maternal serum when available: PrePY, ZOOM Pilot and Flora cohorts).

2. To characterize the differences in the key-pathogen specific antibody levels based on maternal nutritional status.

Cohorts, subjects and samples. Briefly, eligibility criteria and sample collection details for each of the studies are as follows. Two cohorts from Bangladesh were included. The first one leveraged clinical data and biological specimens that were collected under The Hospital for Sick Children Research Ethics Board at icddr,b in Dhaka, Bangladesh from mother-infant pairs as part of the Maternal Vitamin D for Infant Growth (MDIG) trial (#NCT01924013)(1) and the Maternal Vitamin D for ARI in Infancy (MDARI)(2) cohort study (#NCT02388516), which was nested within the MDIG trial. Hand-expressed, mid-feed human milk samples were collected at 3 months postpartum, and frozen in aliquots at approximately -70°C until shipment to The Hospital for Sick Children for storage at -80°C. A total of 834 human milk samples available, of which 246 mothers' samples at 13 weeks were selected based on case-control design to include samples from those mothers with and without positive respiratory outcomes for RSV and influenza A/B, parainfluenza 1-3, metapneumovirus, adenovirus and *Streptococcus pneumoniae* in their infants, verified by qPCR. Maternal blood

samples were collected at 3 months postpartum; samples were centrifuged for 30 to 60 minutes at room temperature and serum was separated into 0.25 mL aliquots, and stored at approximately -70°C in Dhaka until transfer to a -80°C freezer for long term storage at The Hospital for Sick Children. *PROVIDE study* (3,4) (#NCT01375647) was recruited in Dhaka and Mirpur, Bangladesh under IRB approval to assess the impact of enteric inflammation on polio and rotavirus vaccine responses and infant health including respiratory and diarrheal outcomes. Briefly, a birth cohort of children from the Mirpur urban slum in Dhaka, Bangladesh were recruited and followed for 2 years. A total of 700 human milk samples were available at 1 week, of which 256 mothers' samples were selected based on case-control design and included samples from mothers with and those without infants positive for diarrheal illnesses verified by qPCR for *Shigella*, ETEC, *Campylobacter*, rotavirus and *Cryptosporidium* by 1 year of age. Hand-expressed human milk was collected from the mothers in the morning time before noon in the field clinic and were kept in a cool box before frozen in -80°C with 4-6 hours after collection. All samples were only thawed maximum once before use here. The Karachi, Pakistan *PrePY study* is a study of the epidemiology of pertussis in infants (5). Infants were enrolled either at birth (closed cohort) or at ages up to 10 weeks (open cohort) and followed until 18 weeks of age. Nasopharyngeal swab specimens were obtained from infants who met a standardized syndromic case definition and tested for *Bordetella pertussis* using real-time polymerase chain reaction. A total of 156 human milk samples from 49 mothers were available throughout the first 18 weeks. Milk was collected on manual expression, it was brought at 4°C to the laboratory, aliquoted and frozen at 80°C*.* The Peru cohort of the *MAL-ED study* (6) ("Interactions of Enteric Infections and Malnutrition and the Consequences for Child Health and Development", #NCT02441426) included recruitment of mother-infant pairs for microbial carriage and diarrhea data of the children. Human milk samples were serially collected monthly on 36 mothers for the entire period of lactation. This study utilized colostrum and human milk samples until 18 weeks of lactation. Monthly stool collection and longitudinal surveillance detected symptomatic and asymptomatic enteric in fection throughout the surveillance period. The specimens were obtained by manual expression. Children were put to breast and after letdown and a light snack the child was removed from the breast and the milk collected. *Rochester Lifestyle study (ZOOM Pilot)* enrolled generally healthy mothers and their infants who have birth to full-term (>37 weeks of gestation) babies to a cross-sectional study of maternal lifestyle impact on human milk and infant gut microbiome composition in rural and urban/suburban Rochester, NY, U.S. (7,8); only Rochester urban/suburban cohort was included here. Mothers were instructed to collect 30 ml of human milk in the morning at an average of 6 weeks of age. Samples were immediately

frozen in home freezer and transferred frozen to the laboratory and frozen at -80°C, or kept in cold after collection and frozen at -80°C with 4-6 hours after collection. All the 23 samples available were included. *Flora study in Finland* (9) (trial registry number: NCT00298337), 1229 mothers were recruited in the Helsinki suburban area to a large randomized, double blind, placebo-controlled study of pre- and probiotic supplementation and assessment of human milk composition, collected under The ethics committee of the Hospital for Children and adolescents, University of Helsinki, Helsinki, Finland. The lactating mother expressed a 5-20 ml aliquot of BM at the beginning of breast-feeding her infant on days 0 to 3 days postpartum and at 12 weeks. Only samples from non-supplemented mothers were assessed here. Of the 300 colostrum and mature milk samples collected at 12 weeks, 100 samples randomly selected were assessed in this study. The sample was immediately frozen at home and brought frozen to the researchers and hence put into a -70°C freezer. *Cryptosporidium Burden Study* was used as a validation set. A natural history study of cryptosporidiosis in infants was conducted in the Mirpur community of Dhaka, Bangladesh. Children were longitudinally followed from birth to three years of age (National Clinical Trial Identifier: NCT02764918) and enteric infections identified by TAC. Hand-expressed human milk was collected from the mothers before noon in the field clinic and were kept in a cool box before being frozen in -80°C with 4-6 hours after collection. Alltogether 205 samples were available; of those 144 were selected for assays based on a cohort design to include all those mothers with infants with fecal samples positive for rotavirus, *Campylobacter* or *Shigella* infections detected by PCR during the first year of life.

Paired maternal serum was available from 34 mothers in the Peru MAL-ED study and 60 mothers in the MDIG/Bangladesh study. All samples were thawed at most once for aliquoting, then thawed a second time for being assayed. All samples were collected under local IRB protocols.

Selection of antigens for protein arrays. Selection of proteins followed two approaches: 1) an empirical approach utilizing the databases from prior studies performed at Antigen Discovery, Inc. (ADI, Irvine, CA), and 2) a hypothetical approach using *in silico* prediction of antigenic targets and orthologues of confirmed antigenic targets already identified in ADI databases. The first approach was used for pathogen proteins printed on the Gen 1 Chip (**Table 1**) and has been described (10). Proteins were selected for inclusion based on seroprevalence rates and correlation with exposure to pathogens, or where limited data were available, homology with other antigens. For pathogen proteins that were

printed on the Gen 2 Chip (**Table 1**), the second approach was used for protein selection, with the exception of Human Adenovirus, Rotavirus A, and RSV, where the whole proteome was printed. For *Campylobacter jejuni*, *Cryptosporidium* spp., *Vibrio cholerae*, *Acinetobacter baumannii*, and Group B *Streptococcus*, proteins were selected based on a prioritization scheme that consisted of ranking of proteins by scores for protein features that predict surface localization and secretion. Methods used for scoring included 1) major antigen families identified in literature search, 2) prediction of transmembrane domains using TMHMM 2.0 (11), 3) prediction of signal peptides using SigalP 5.0 (12), 4) prediction of subcellular localization using PSORTb 3.0.2 (13) with priority from highest to lowest for: outer membrane, extracellular/secreted, plasma membrane and periplasmic, 5) gene ontology word search (14) with the following search terms: Flagellin, flagella, flagellar, Fli, fimbrial, flag Membrane, secreted, adhesin, cell wall, surface, hemagglutinin, transport Cilia, pilus, pili, Pil, porin, holin, Ton Omp, receptor, transfer protein Toxin, antigen, trigger factor, protease, lysin and adhesin. For *Klebsiella pneumoniae*, a more detailed bioinformatics analysis was performed. Bioinformatic analysis was based on a manually collated collection of known surface protein domains (including porins, autotransporter, secretion systems, fimbrial tips, Ig-like domains, intimin-invasin) combined with signal peptide predictions. Collated Pfam domains were used to expand the collection and include DUFs (domain of unknown functions) by using the annotated Pfam CLANS (groups of related Pfam domains, manually derived and available in Pfam) as well as the HHPRED predictions of related domains available in Pfam (v31)(15) for selection of domains likely to encode surface-exposed proteins or protein domains. Emphasis was based on proteins conserved in several genomes of the species, which was selected by comparison with several publicly available genome sequences.

Creation of arrays, including balancing factors. Proteome microarrays were fabricated by using a library of partial or complete open reading frames (ORFs) cloned into a T7 expression vector pXI that has been established at ADI. Briefly, the clone library was created through an *in vivo* recombination cloning process with PCR-amplified coding sequences, and a complementary linearized expressed vector transformed into chemically competent *E. coli* cells was amplified by PCR and cloned into the pXI vector using a high-throughput PCR recombination cloning method as described in detail elsewhere (16). All 783 clones were sequenced (Retrogen, Inc., San Diego, CA), and the results matched the correct target for all clones. Proteins were expressed using an *E. coli in vitro* transcription and translation (IVTT) system (Rapid

Translation System, 5 Prime, Gaithersburg, MD, U.S.). Each expressed protein includes a 5' polyhistidine epitope tag and a 3' hemagglutinin (HA) epitope tag. After expressing the proteins according to the manufacturer's instructions, translated proteins were printed onto nitrocellulose-coated glass AVID slides (Grace Bio-Labs, Inc., Bend, OR) using an OmniGrid accent robotic microarray printer (Digilabs, Inc., Marlborough, MA). Each slide contained eight nitrocellulose pads on which the full array was printed (this allowed eight samples to be probed per slide using sealed chambers that isolate the arrays). In addition to the targeted proteins, IVTT reactions without expression insert were included and spotted in replicates on each subarray of each pad. These "IVTT controls" served as a normalization factor for array-to-array variation. Purified recombinant proteins commercially sourced or provided by collaborators were printed in two concentrations: 0.1 and 0.03 mg/mL. Microarray chip printing and protein expression were quality checked by probing random slides with anti-His and anti-HA monoclonal antibodies with fluorescent labeling.

Probing details. Milk samples were diluted 1:5 in a 1.5 mg/mL *E. coli* lysate solution (Antigen Discovery, Inc., Irvine, CA) in protein arraying buffer (Maine Manufacturing, Sanford, ME) and incubated at room temperature for 30 min. Microarray slides were hydrated in arraying buffer and then probed with 250 µL of the preincubated milk samples using sealed, fitted slide chambers to avoid cross-contamination between arrays. Arrays were incubated overnight at 4°C with agitation, washed three times with Tris-buffered saline (TBS)-0.05% Tween 20 (Thermo Scientific, J77500K8, diluted 20x in molecular grade water), and incubated with Cy3-conjugated anti-human IgG diluted 1:200 in arraying buffer and biotin-conjugated anti-Human IgA diluted 1:1000 (Jackson ImmunoResearch, West Grove, PA). Arrays were washed three times with TBS–0.05% Tween 20 and incubated with streptavidin-conjugated SureLight P-3 (Columbia Biosciences, Frederick, MD) at room temperature, protected from light. Arrays were washed 3x with TBS–0.05% Tween 20, 3x with TBS, and once with water and then air dried by being centrifuged at 1,000 x g for 4 min and left overnight in a desiccator before scanning. Probed microarrays were scanned using a GenePix 4300A high-resolution microarray scanner (Molecular Devices, Sunnyvale, CA), and an image file (.tiff) was saved for each array using GenePix pro 7 software. The signals in the scanned images were quantified using Mapix software (Innopsys). All further data processing was performed in R (http://www.R-project.org). Data were normalized by first transforming raw values using the base 2 logarithm. Next, the data set was normalized to remove systematic effects by subtracting the median signal intensity of

the IVTT control spots for each sample. Since the IVTT control spots carry not only the chip, sample, and batch-level systematic effects, but also antibody background reactivity to the IVTT system, this procedure normalizes the data and provides a relative measure of the specific antibody binding versus the nonspecific antibody binding to the IVTT controls. With the normalized data, a value of 0.0 means that the intensity is no different than that of the IVTT controls, and a value of 1.0 indicates a doubling with respect to IVTT control spots. IgA and IgG normalized data were analyzed separately, due to independent normalized intensity scales.

Statistical Analysis Plan. Briefly, protein seropositivity was defined for each antigen as twice the sample-specific (array-level) median IVTT background, or a normalized signal of 1.0. Antibody breadth was calculated as the number of seropositive protein responses for an individual sample. Since the proteins on the multipathogen chips were a downselection and not representative of full proteomes, antibody breadth scores were defined as the proportion of seropositive antigen-specific responses per pathogen, rather than count of seropositive responses, and were calculated for each individual sample. A reactive protein was defined as an antigen with median normalized signal of 1.0 across all study subjects, i.e. at least 50% of the study population had a seropositive response—to account for longitudinal sampling, only the maximum value of each antigen was used for each subject. The set of IgA- or IgG-reactive antigens were used in certain analyses to reduce the number of false discoveries. Summary statistics included mean and median breadth scores, and 95% confidence intervals and interquartile ranges. Antibody magnitude was analyzed as the continuous normalized signal intensity data. Where applicable, analyses of protein responses were stratified by pathogen and analyses of subjects by study cohort or economic region. Comparisons of mean signals between longitudinal time points was done using paired T tests. Independent comparisons of mean signals between economic region or between cohorts used unpaired T tests. Comparison of antibody breadth scores was done using Wilcoxon's rank sum tests. Counts of antigens recognized by human milk and serum IgA and IgG were displayed in upset plots using the UpSetR package in R (17). Data dimensional reduction was performed by principal component analysis (PCA), and PC values between economic regions was tested using T tests. The associations of biological and environmental covariates with all antibody concentrations was modeled using multivariable linear mixed effects regression (LMER) with the lme4 package in R (18) to control for multiple antibody measurements for each sample. Models were fit with noramlized

signal intensity of all antigens as the dependent variable and included study cohort or economic classification (LMIC or HIC), maternal age (yr), parity, highest education level (ordinal variable), BMI and infant feeding pattern as predictor variables and random intercepts for study subjects. Only mature breastmilk samples that were collected between 6 and 18 weeks postpartum were included in the models. Some cohorts had high proportions of missing data or no data for certain covariates and were excluded. Therefore, multiple models were fit to obtain covariate effect estimates with maximal sample size. Associations with antibody concentrations against individual antigens was modeled using ordinary least squares regression with the same covariates as the LMER models. Again, multiple models were fit excluding cohorts with missing data for certain covariates. All coefficients reported were returned from models fit using restricted maximum likelihood (REML). To generate P-values for LMER models, the models were refit using maximum likelihood (ML) and compared by ANOVA against null models with the coefficient removed using ML. To assess the effect of specific IgA responses on likelihood of subsequent enteric or respiratory illness, multivariable logistic regression models were fit to cases of mothers with infants that developed disease and controls of mothers with infants that did not develop disease. Detailed follow-up of infant enteric outcomes was available for the PROVIDE cohort in Bangladesh (human milk samples collected at approximately 1 week postpartum), and respiratory outcomes were available for the MDIG cohort in Bangladesh (human milk samples collected at 3 months postpartum). Therefore, the samples from the two Bangladesh cohorts were used in the logistic regression models for enteric and respiratory illness, respectively. The logistic regression models were adjusted by coviarates maternal age, parity and BMI. For four of 246 samples in the MDIG Bangladesh cohort, missing BMI data were imputed using the within-cohort median. To assess the effect of specific IgA responses on risk of infant infection with enteric pathogens, the PROVIDE cohort antibody responses to each enteric pathogen antigen were classified into top half and bottom half of responses and modeled using Cox proportional hazards models on time to first PCR-confirmed infection with the corresponding pathogen. Kaplan-Meier plots were used to show survival curves for each pathogen. Log-rank test P-values were used for univariate models, and multivariable Cox model P-values were used to for models adjusted by sex of infant, maximum number of days of exclusive breastfeeding, length for age of infant at enrollment, parity, maternal age, maternal BMI, maternal education (in years), household income, household expenditure and ordinal categories of drinking water treatment (1 = none, 2 = stand and settle, 3 = water filter, 4 = solar disinfection, 5 = boil, 6 = cloth strain, 7 = bleach/chlorine treatment). Only mothers with children with PCR-confirmed infection to specific pathogens during the first year of life were included in

corresponding Cox models to control for lack of exposure. Cox models were also performed on enteric illness among PCR-confirmed cases, defined as an attributable fraction score (AFE) indicating causality of enteric illness. Cox models for both infections and enteric illness were also performed on the whole PROVIDE cohort irrespective of PCR-confirmed infant infections. To validate our findings in the Cox regression models, samples tested from the Cryptosporidium Burden Study were analyzed in the same was as the PROVIDE study with the exception of adjusting coviariates, which included the following: maternal age, maternal BMI, household income, household expenses, water treatment, drain access and HAZ score of infants at birth. All P-values were adjusted for the false discovery rate (19).

Supplementary Figures

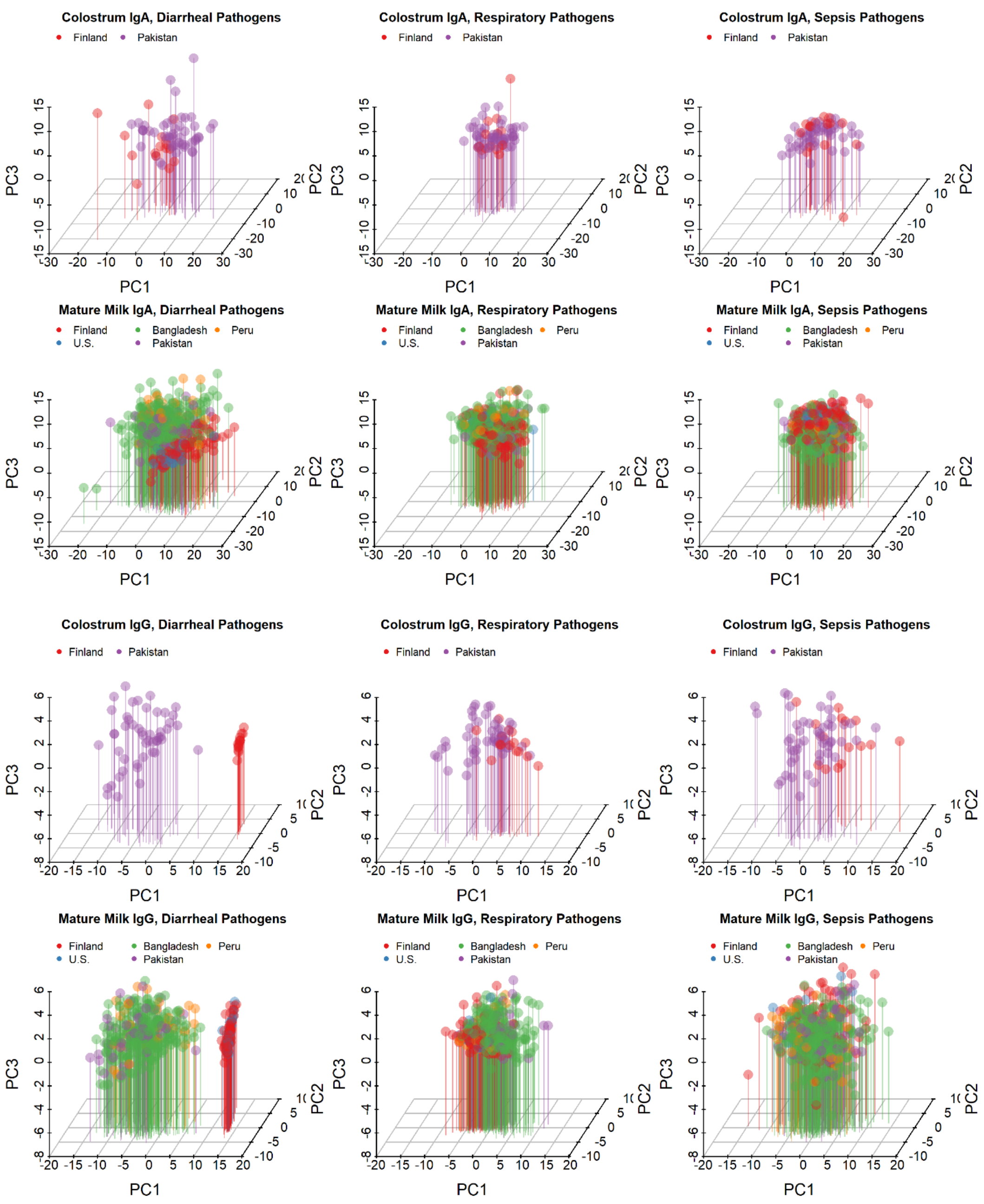

Figure S1. Principal component analysis by geographic region for colostrum and mature human milk IgA and IgG. Principal component analysis was performed on colostrum and mature human milk IgA responses to enteric, respiratory and sepsis pathogens independently. The three dimensional plots show principal component values for each individual as points colored by geographic region. The top row of plots show colostrum IgA responses, for which samples were available from Finland and Pakistan. The bottom row of plots show mature milk IgA responses, for which samples were available from Finland, the U.S. (Rochester, NY), Bangladesh, Pakistan and Peru. Enteric, respiratory and sespis pathogens are arranged in columns 1, 2 and 3, respectively. Notably, the samples selected from the Bangladesh MDIG cohort could bias differential analysis of antibody concentrations to influenza A and B and RSV due to the case-control design, which may not reflect reflect population prevalences of influenza and RSV. However, case sample selection was not related to other respiratory pathogens such as pneumococcus, *Mtb*, and pertussis, which formed the majority of specific antibody responses among respiratory pathogens. Indeed, exclusion of influenza A and B and RSV from the PCA plots had negligible effect on differential profiles and significance levels. Likewise, exclusion of the Bangladesh MDIG samples yielded the same PCA results with higher but still significant P-values due to the smaller sample size.

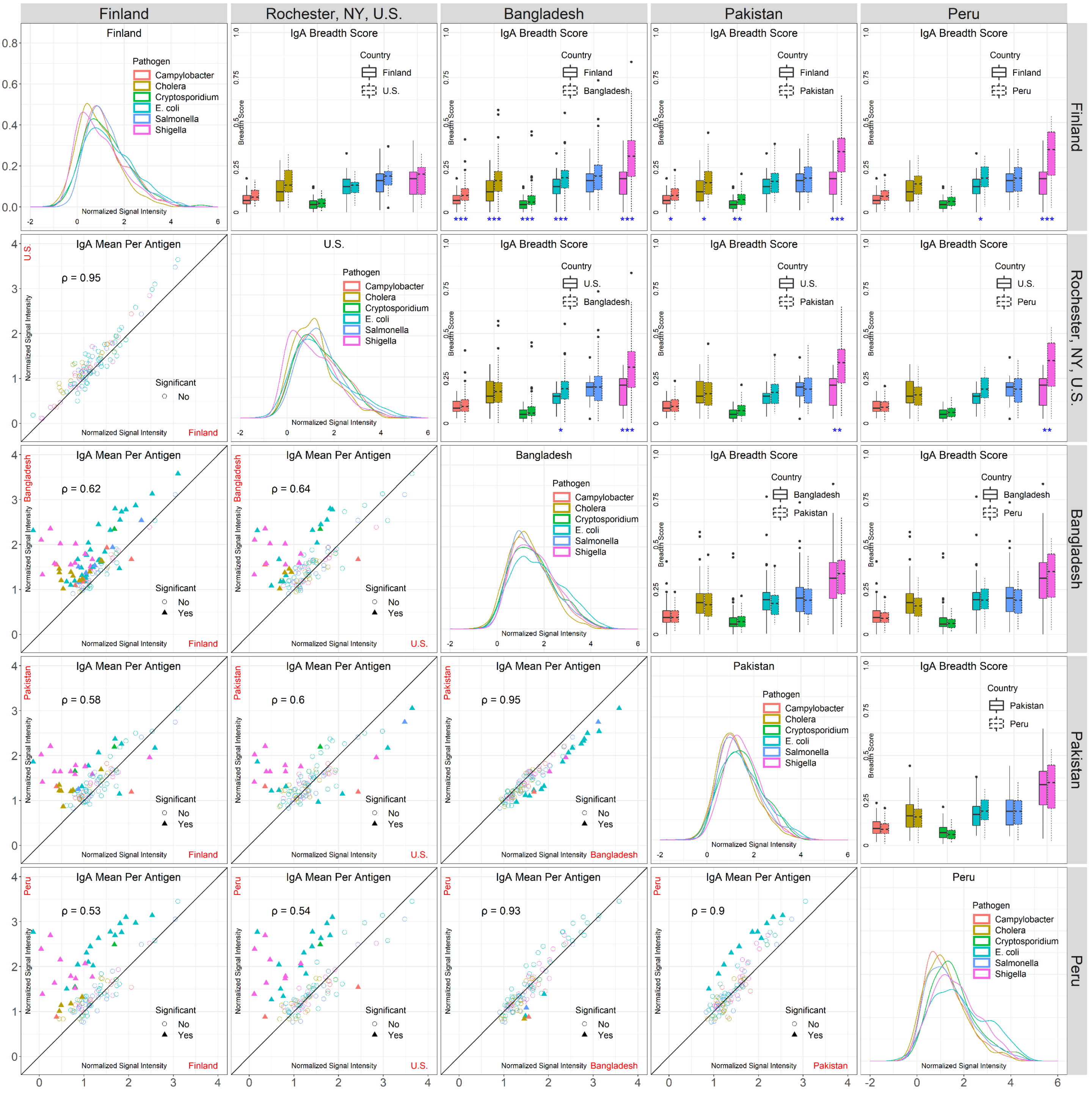

Figure S2. Differential analysis of enteric pathogen-specific mature human milk IgA by geographic region. The matrix plot shows summarized antibody distribution data for each geographic region. The density plots on the diagonal of the matrix show the distribution of normalized signal intensities for each of the primary enteric pathogens. *E. coli*: diarrheagenic types EAEC, EPEC and ETEC. Rotavirus and Adenovirus 40/41 were omitted due to low numbers of reactive antigens. The color keys on the density plots also apply to the lower-half scatter plots and the upper-half boxplots. The upper-half plots show pair-wise comparisons of IgA breadth scores, defined as the proportion of seropositive (normalized signal ≥ 1.0) antigens per pathogen for each individual. The x-axes show each color-coded enteric pathogen grouped by country (indicated by solid or dashed box outlines). The y-axes show the IgA breadth scores with the boxes representing the median and interquartile range. Significant cohort differences by Wilcoxon's rank sum tests are shown by blue asterisks below each pathogen. * ≤ 0.05, ** ≤ 0.005, *** ≤ 0.0005. The lower-half plots show pair-wise comparisons of mean IgA concentration for each of the reactive antigens from enteric pathogens. Each

point represents the mean normalized signal intensity for an individual antigen, colored by pathogen, and solid triangles represent antigens with significant differential reactivity between cohorts by T tests after correction for the false discovery rate. For ease of viewing, the geographic regions applying to each axis are labeled in red text. Additionally, each plot includes a Pearson's correlation coefficient (ρ).

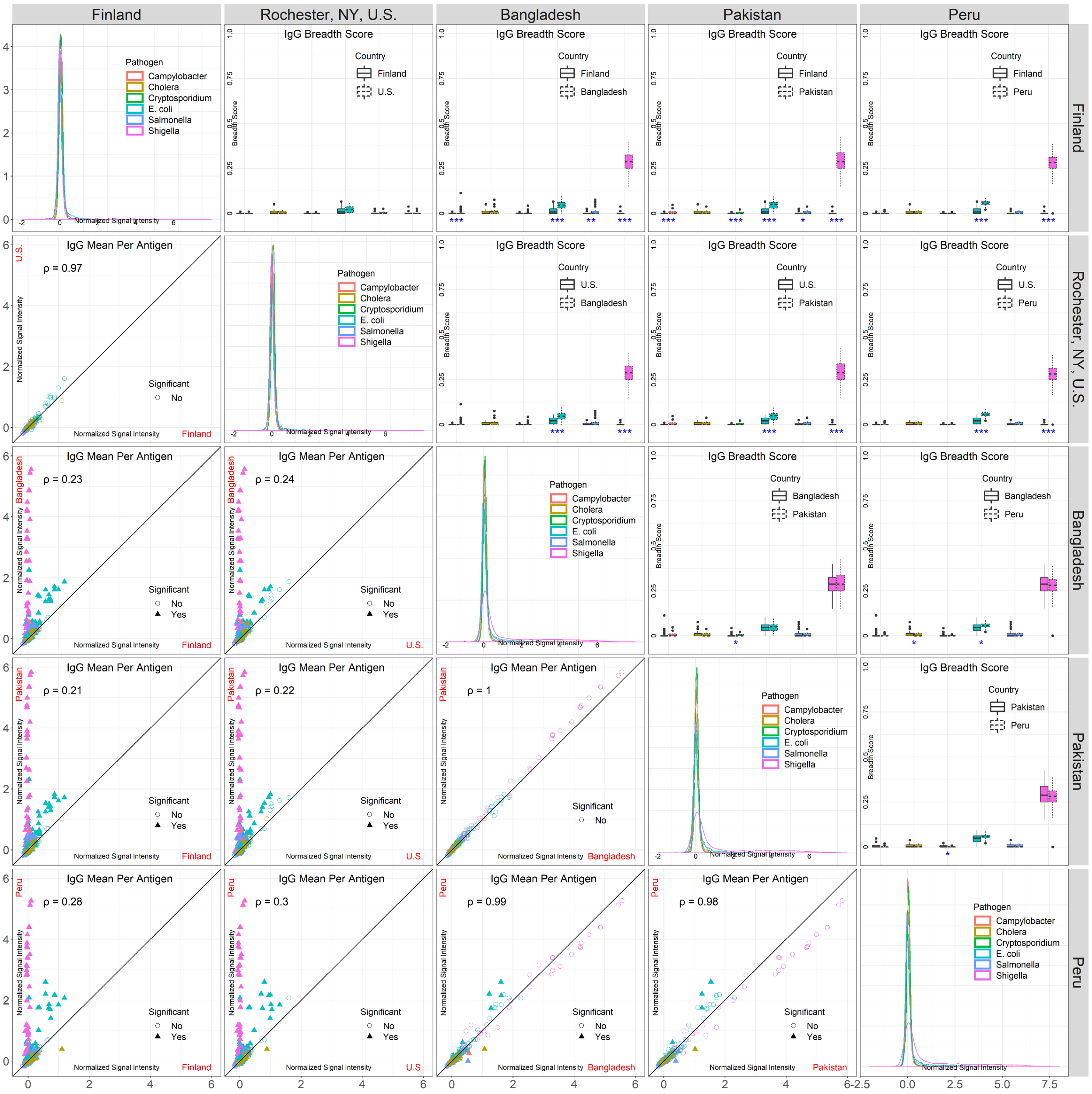

Figure S3. Differential analysis of enteric pathogen-specific mature human milk IgG by geographic region.
The matrix plot shows summarized antibody distribution data for each geographic region in a matrix format. The density plots on the diagonal of the matrix show the distribution of normalized signal intensities for each of the primary enteric pathogens. *E. coli*: diarrheagenic types EAEC, EPEC and ETEC. Rotavirus and Adenovirus were omitted due to low numbers of reactive antigens. The color keys on the density plots also apply to the lower-half scatter plots and the upper-half boxplots. The upper-half plots show pair-wise comparisons of IgA breadth scores, defined as the proportion of seropositive (normalized signal ≥ 1.0) antigens per pathogen for each individual. The x-axes show each color-coded enteric pathogen grouped by country (indicated by solid or dashed box outlines). The y-axes show the IgA breadth scores with the boxes representing the median and interquartile range. Significant cohort differences by Wilcoxon's rank sum tests are shown by blue asterisks below each pathogen. * ≤ 0.05, ** ≤ 0.005, *** ≤ 0.0005. The lower-half plots show pair-wise comparisons of mean IgG concentration for each of the proteins from enteric pathogens. Each point represents the mean normalized signal intensity for an individual antigen, colored by pathogen, and solid triangles represent antigens with significant differential reactivity between cohorts by T tests after correction for the false

discovery rate. For ease of viewing, the geographic regions applying to each axis are labeled in red text. Additionally, each plot includes a Pearson's correlation coefficient (ρ).

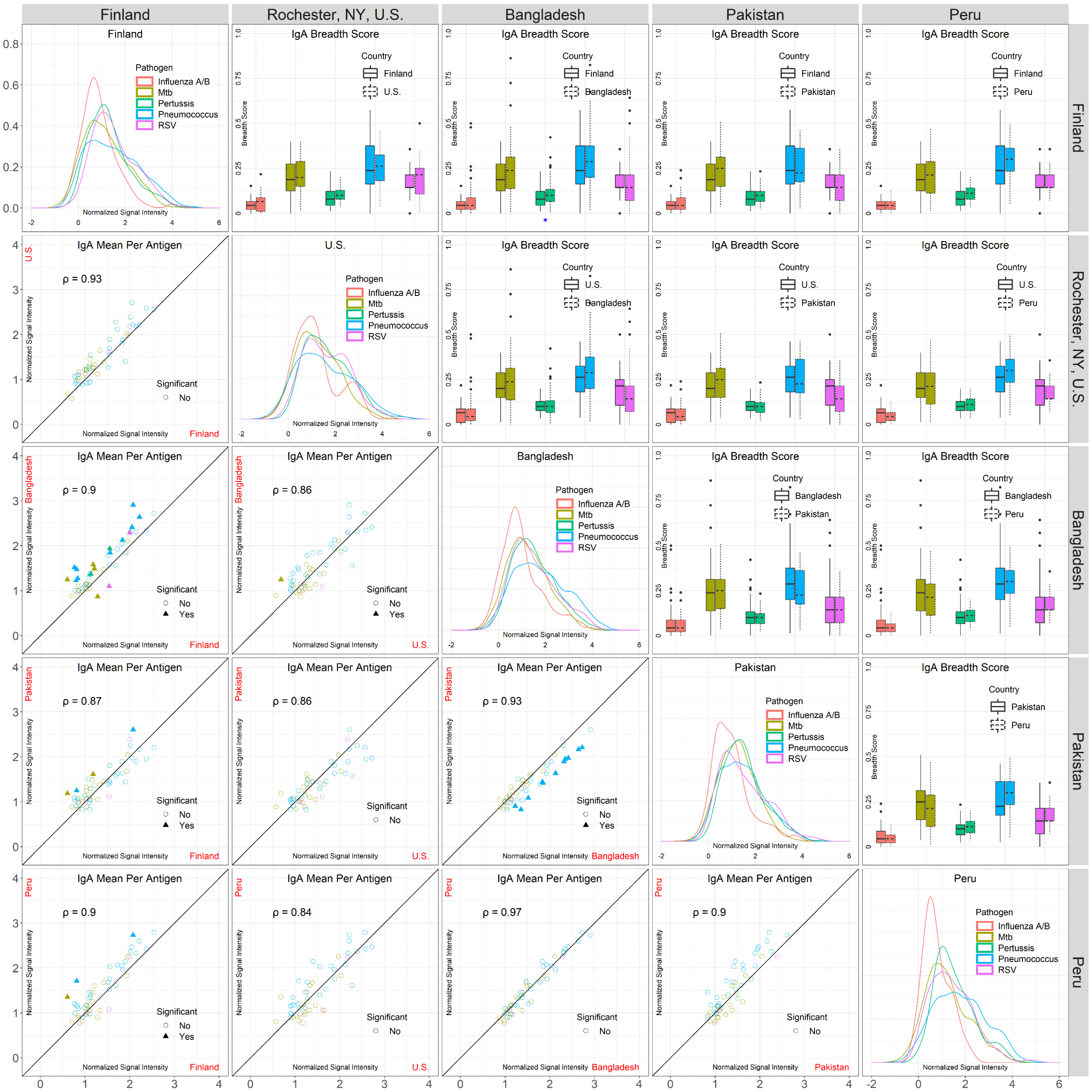

Figure S4. Differential analysis of respiratory pathogen-specific mature human milk IgA by geographic region. The matrix plot shows summarized antibody distribution data for each geographic region in a matrix format. The density plots on the diagonal of the matrix show the distribution of normalized signal intensities for each of the primary respiratory pathogens. The color keys on the density plots also apply to the lower-half scatter plots and the upper-half boxplots. The upper-half plots show pair-wise comparisons of IgA breadth scores, defined as the proportion of seropositive (normalized signal ≥ 1.0) antigens per pathogen for each individual. The x-axes show each color-coded respiratory pathogen grouped by country (indicated by solid or dashed box outlines). The y-axes show the IgA breadth scores with the boxes representing the median and interquartile range. Significant cohort differences by Wilcoxon's rank sum tests are shown by blue asterisks below each pathogen. * ≤ 0.05, ** ≤ 0.005, *** ≤ 0.0005. The lower-half plots show pair-wise comparisons of mean IgA concentrations geographic regions for each of the reactive antigens from respiratory pathogens. Each point represents the mean normalized signal intensity for an individual antigen, colored by pathogen, and solid triangles represent antigens with significant differential reactivity between cohorts by T tests after correction for the false discovery rate. For ease of viewing, the geographic regions applying to each axis are labeled in red text. Additionally, each plot includes a Pearson's correlation coefficient (ρ).

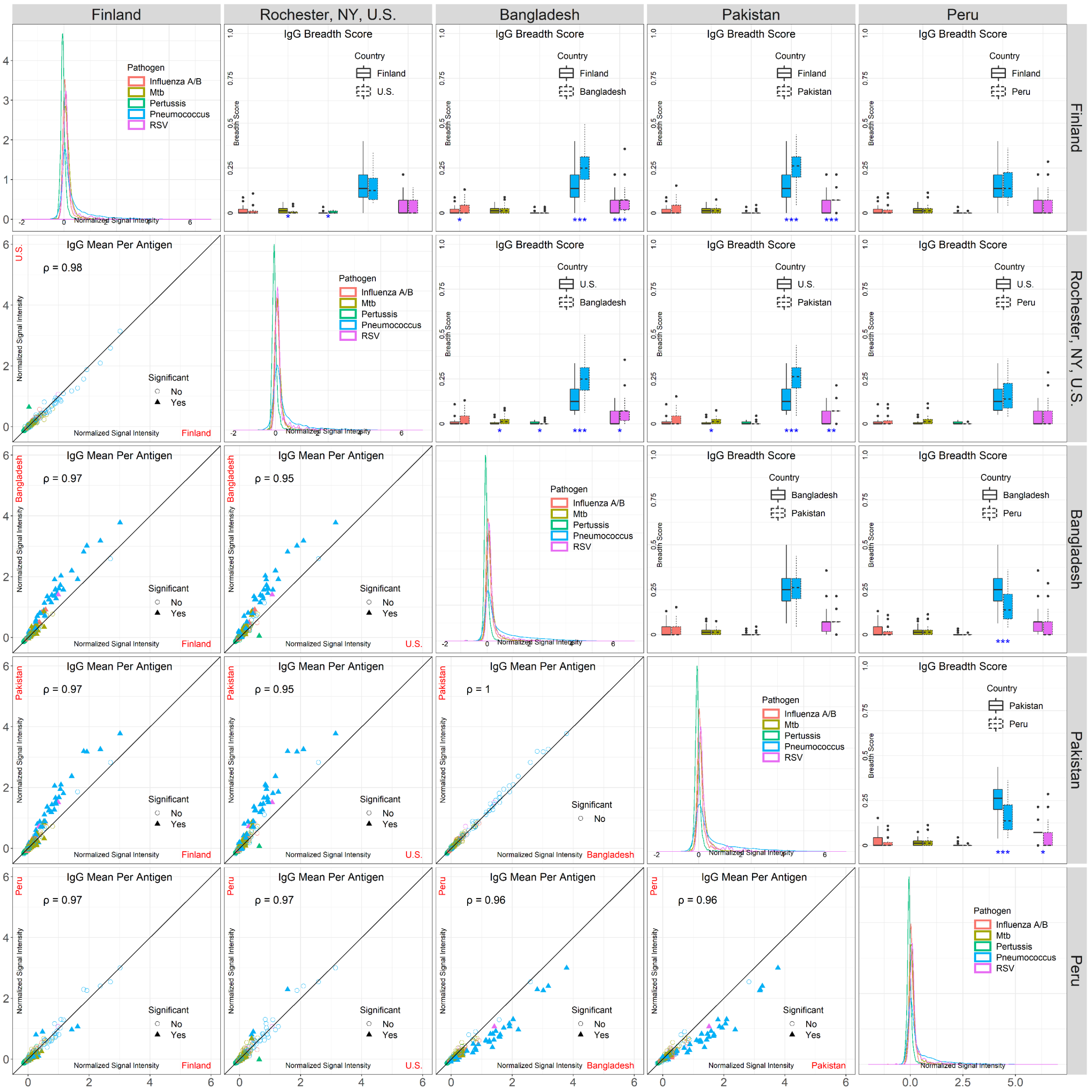

Figure S5. Differential analysis of respiratory pathogen-specific mature human milk IgG by geographic region. The matrix plot shows summarized antibody distribution data for each geographic region in a matrix format. The density plots on the diagonal of the matrix show the distribution of normalized signal intensities for each of the primary respiratory pathogens. The color keys on the density plots also apply to the lower-half scatter plots and the upper-half boxplots. The upper-half plots show pair-wise comparisons of IgA breadth scores, defined as the proportion of seropositive (normalized signal ≥ 1.0) antigens per pathogen for each individual. The x-axes show each color-coded respiratory pathogen grouped by country (indicated by solid or dashed box outlines). The y-axes show the IgA breadth scores with the boxes representing the median and interquartile range. Significant cohort differences by Wilcoxon's rank sum tests are shown by blue asterisks below each pathogen. * ≤ 0.05, ** ≤ 0.005, *** ≤ 0.0005. The lower-half plots show pair-wise comparisons of mean IgG concentrations for each of the reactive antigens from respiratory pathogens. Each point represents the mean normalized signal intensity for an individual antigen, colored by pathogen, and solid triangles represent antigens with significant differential reactivity between cohorts by T tests after correction for the false discovery rate. For ease of viewing, the geographic regions applying to each axis are labeled in red text. Additionally, each plot includes a Pearson's correlation coefficient (ρ).

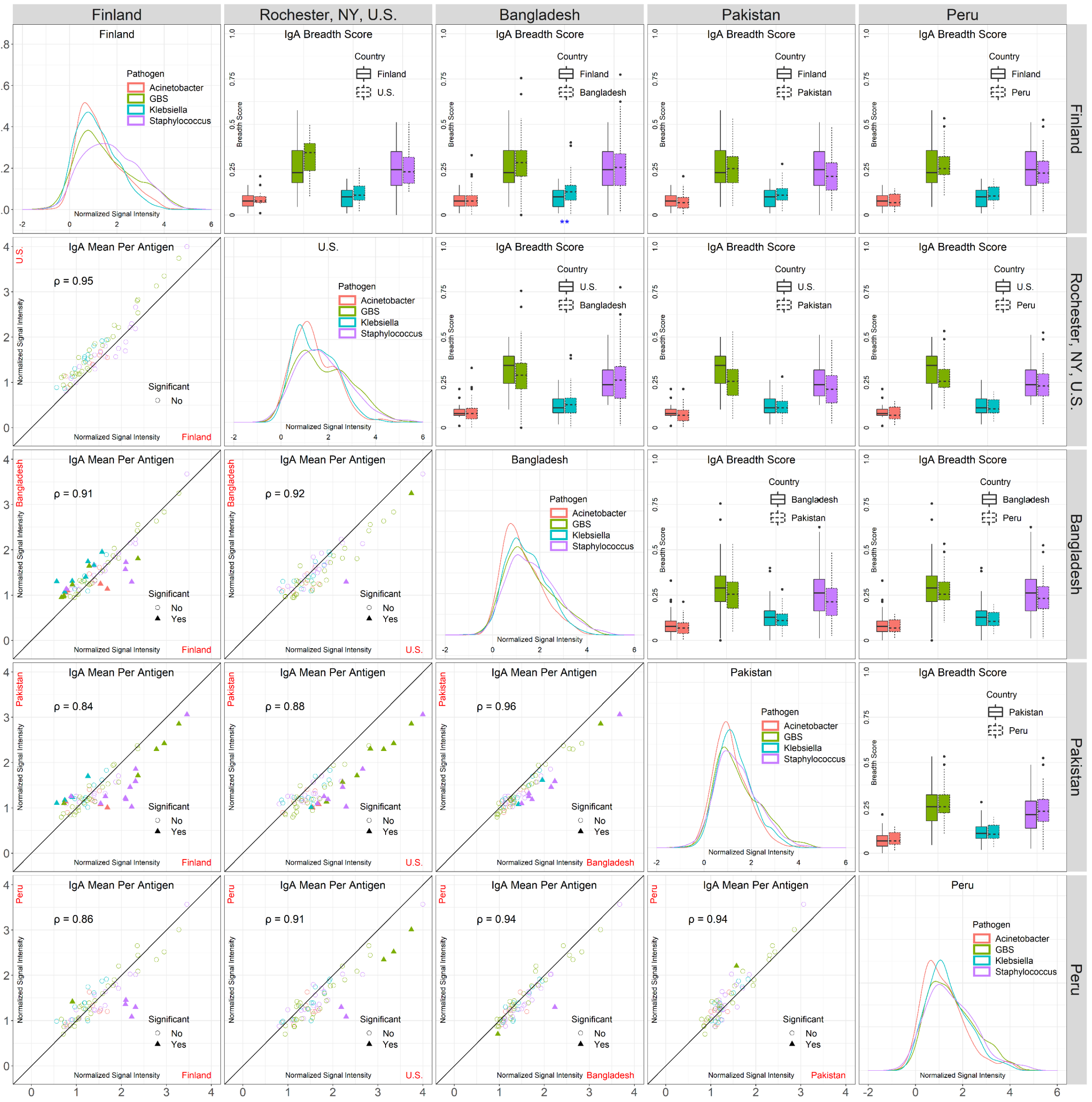

Figure S6. Differential analysis of sepsis-related pathogen-specific mature human milk IgA by geographic region. The matrix plot shows summarized antibody distribution data for each geographic region in a matrix format. The density plots on the diagonal of the matrix show the distribution of normalized signal intensities for each of the primary sepsis pathogens. The color keys on the density plots also apply to the lower-half scatter plots and the upper-half boxplots. The upper-half plots show pair-wise comparisons of IgA breadth scores, defined as the proportion of seropositive (normalized signal ≥ 1.0) antigens per pathogen for each individual. The x-axes show each color-coded sepsis-related pathogen grouped by country (indicated by solid or dashed box outlines). The y-axes show the IgA breadth scores with the boxes representing the median and interquartile range. Significant cohort differences by Wilcoxon's rank sum tests are shown by blue asterisks below each pathogen. * ≤ 0.05, ** ≤ 0.005, *** ≤ 0.0005. The lower-half plots show pair-wise comparisons of mean IgA concentrations for each of the reactive antigens from sepsis pathogens. Each point represents the mean normalized signal intensity for an individual antigen, colored by pathogen, and solid triangles represent antigens with significant differential reactivity between cohorts by T tests after correction for the false discovery rate. For ease of viewing, the geographic regions applying to each axis are labeled in red text. Additionally, each plot includes a Pearson's correlation coefficient (ρ).

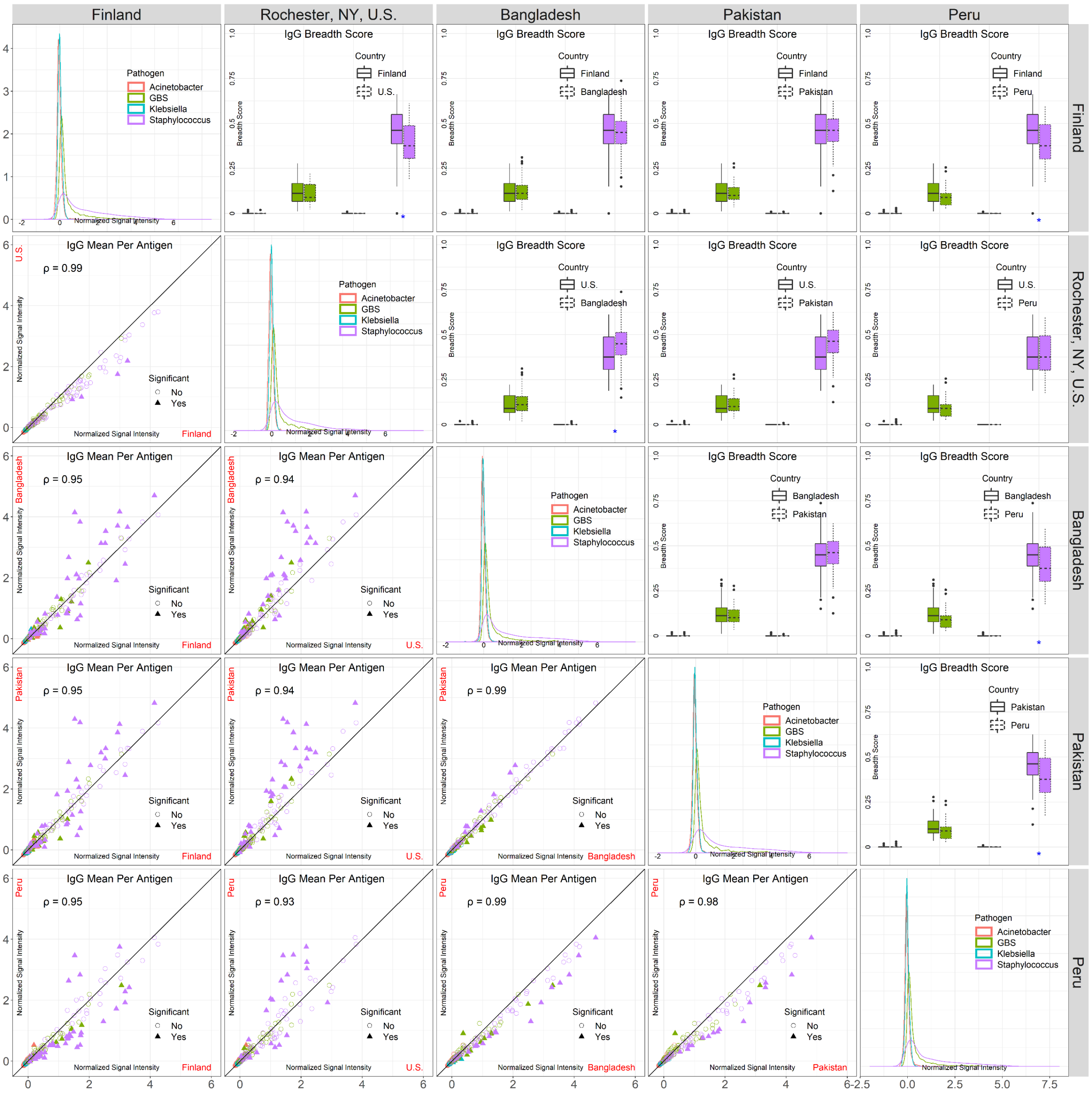

Figure S7. Differential analysis of sepsis-related pathogen-specific mature human milk IgG by geographic region. The matrix plot shows summarized antibody distribution data for each geographic region in a matrix format. The density plots on the diagonal of the matrix show the distribution of normalized signal intensities for each of the primary sepsis pathogens. The color keys on the density plots also apply to the lower-half scatter plots and the upper-half boxplots. The upper-half plots show pair-wise comparisons of IgA breadth scores, defined as the proportion of seropositive (normalized signal ≥ 1.0) antigens per pathogen for each individual. The x-axes show each color-coded sepsis-related pathogen grouped by country (indicated by solid or dashed box outlines). The y-axes show the IgA breadth scores with the boxes representing the median and interquartile range. Significant cohort differences by Wilcoxon's rank sum tests are shown by blue asterisks below each pathogen. * ≤ 0.05, ** ≤ 0.005, *** ≤ 0.0005. The lower-half plots show pair-wise comparisons of mean IgG concentrations for each of the reactive antigens from sepsis pathogens. Each point represents the mean normalized signal intensity for an individual antigen, colored by pathogen, and solid triangles represent antigens with significant differential reactivity between cohorts by T tests after correction for the false discovery rate. For ease of viewing, the geographic regions applying to each axis are labeled in red text. Additionally, each plot includes a Pearson's correlation coefficient (ρ).

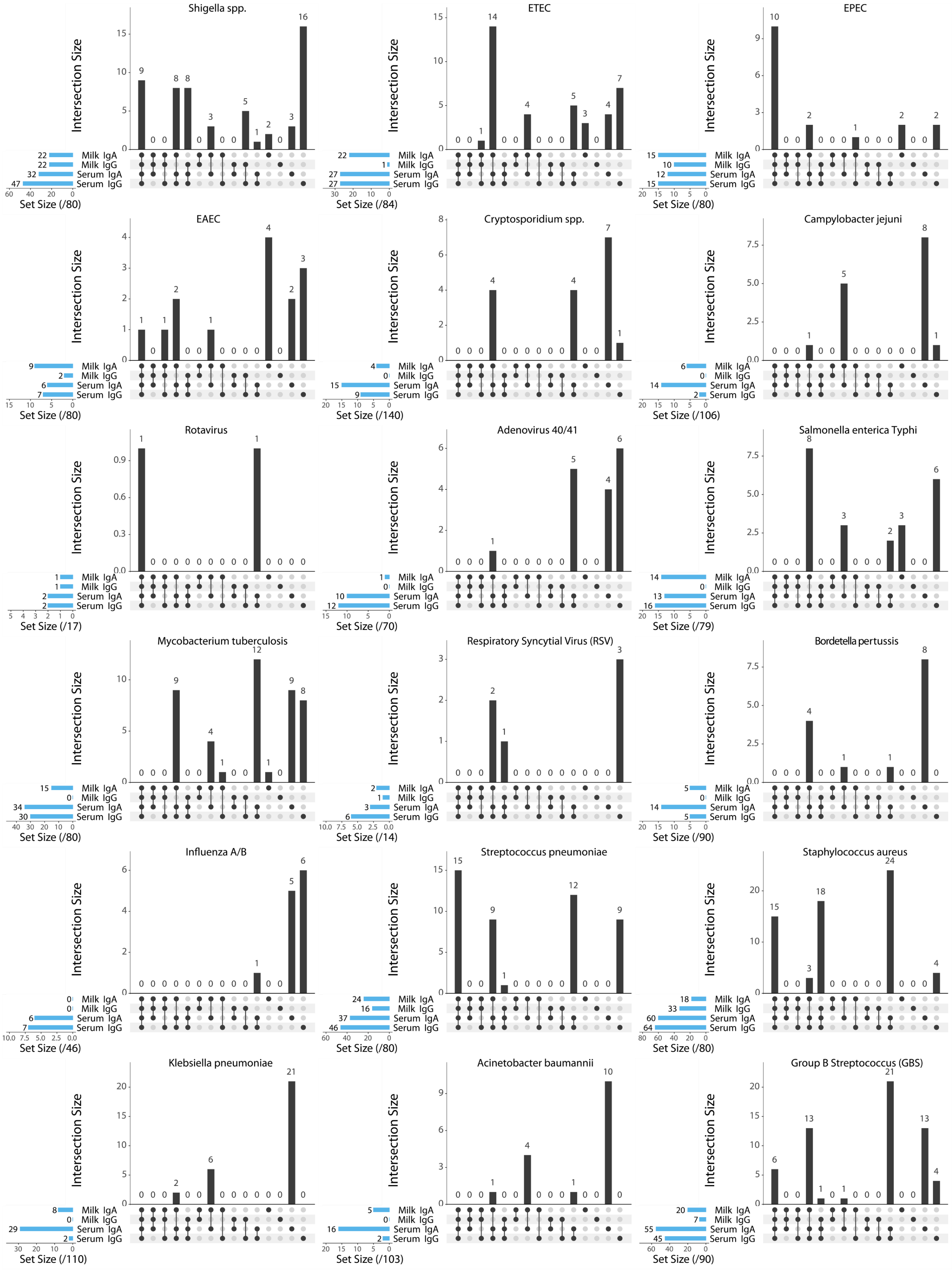

Figure S8. Antigen-specific recognition of IgA and IgG within commonly reactive pathogens in human milk and serum. The "upset" plots show the overlap of antigen reactivity within each pathogen by biological specimen type and antibody isotype. Each combination of categories (milk IgA, milk IgG, serum IgA and serum IgG) is a "set". The vertical bar graph represents the intersections, i.e. count of antigens with overlapping antibody responses between sets. The overlapping

sets are shown below by connected dots. Each set also has a horizontal bar graph with blue bars indicating the total number of reactive antigens identified in each set. Reactive antigens were defined as antigens with median IgA concentrations of at least 1.0 in normalized signal intensity. Each plot has a header indicating the pathogen for which reactive antigens were counted. The intersection bars are ordered by "degree", with the largest number of intersecting sets shown to the left. Only samples from the Peru (n=33 subjects) and Bangladesh (MDIG, n=60 subjects) cohorts, which had paired serum and human milk samples, were included in the upset analysis.

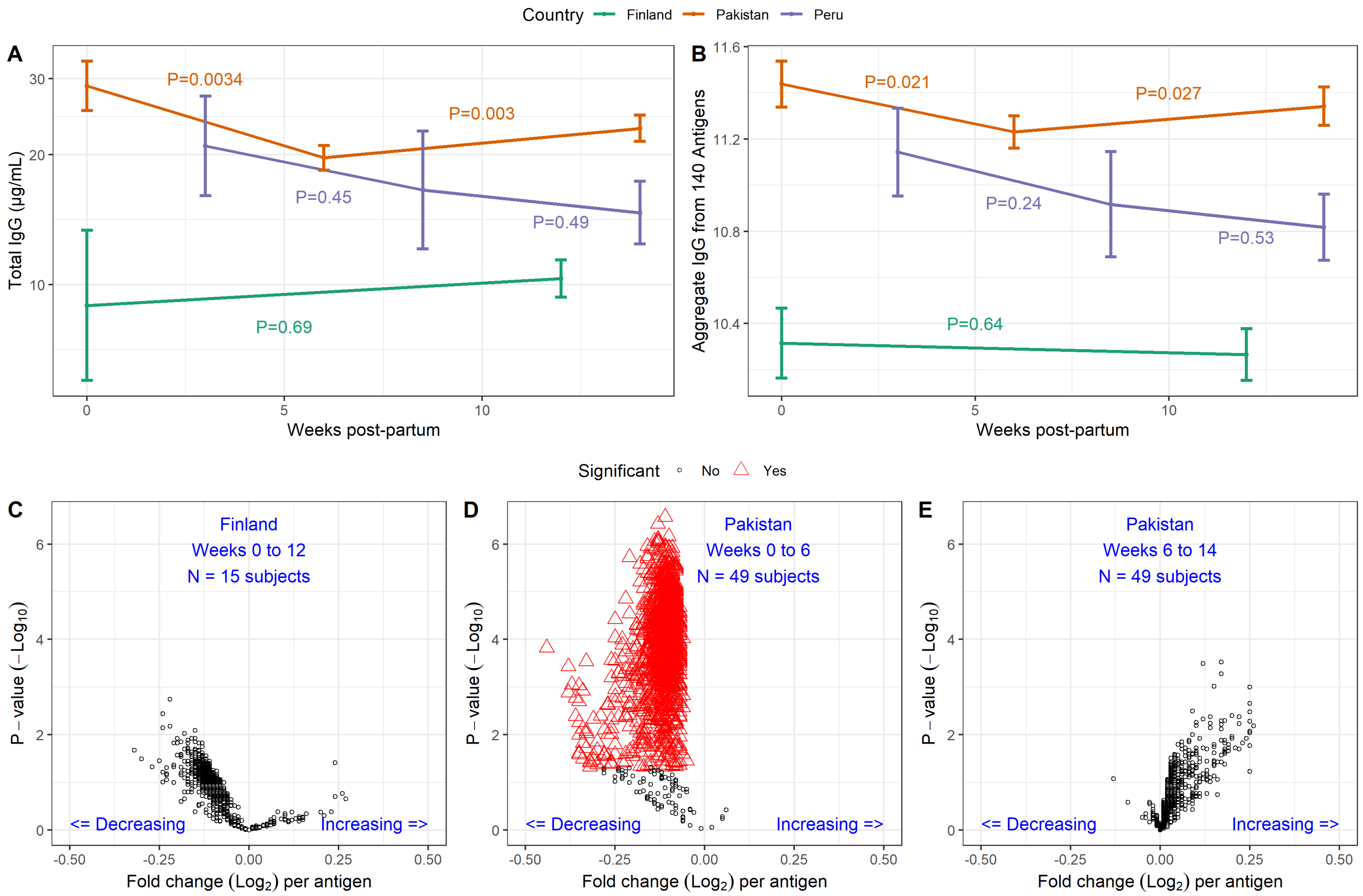

Figure S9. Total IgG and pathogen-specific IgG concentrations decline from colostrum to mature human milk. A-B) The line plots show (A) μg/mL of total IgG in human milk and (B) the mean normalized signal intensity of IgG antibodies specific for 140 reactive pathogen antigens on the multipathogen protein microarray over 12 to 18 weeks postpartum. Three countries with longitudinal sampling are shown: Finland (green), Pakistan (red) and Peru (purple). The vertical bars represent the standard error of the mean. Paired T-test P-values are shown between time points and colored according to cohort. C-E) The volcano plots show the difference between pathogen-specific IgG concentrations between time points for (C) Finland and (D-E) Pakistan. Comparison of samples from Peru is not shown due to low number of week 0 colostrum samples (n = 3). Each black open circle represents an antigen on the multipathogen protein microarray, and red open triangles represent IgG responses to individual antigens that are significant after correction for the false discovery rate. The x-axes show mean differences between time points, and the y-axes show the inverse Log_{10} P-value from paired T-tests.

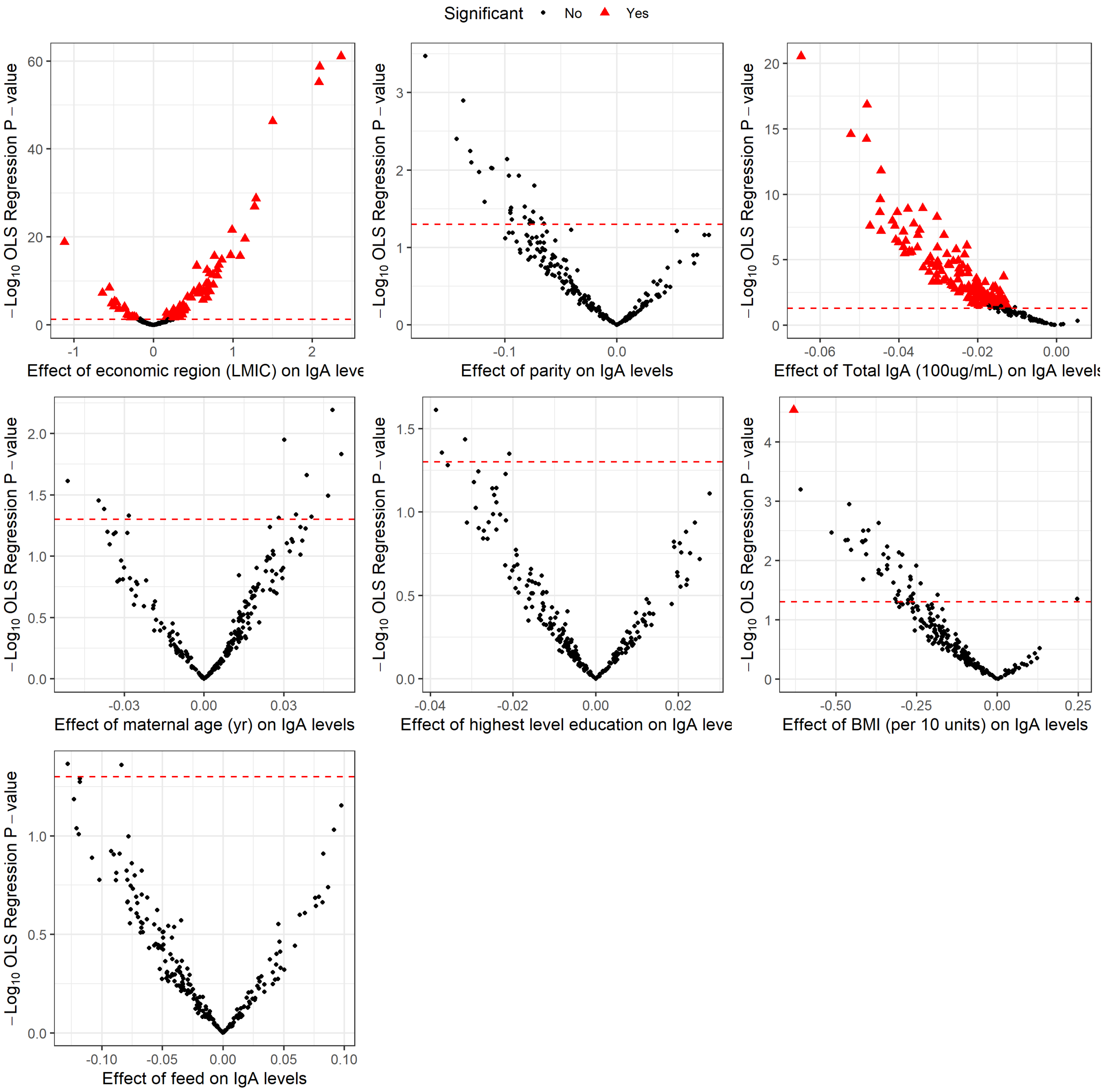

Figure S10. Effect of maternal characteristics on specific IgA antibody concentrations. The volcano plots show the effect estimates from ordinary least squares (OLS) regression models of each reactive antigen on the multipathogen protein microarray (points). The x-axis shows regression coefficients, and the y-axis shows the inverse log_{10} p-values. The red dashed line is drawn at an unadjusted p = 0.05. Antigens with signficant IgA and predictor associations after correction for the false discovery rate are shown in red triangles. Each multivariable model was fit to cohorts with complete predictor data sets or minimal missing values (see Methods). For reference, each -1.0 unit effect estimate represents a 50% decrease in IgA concentrations, and each 1.0 unit effect estimate represents a doubling of IgA concentrations.

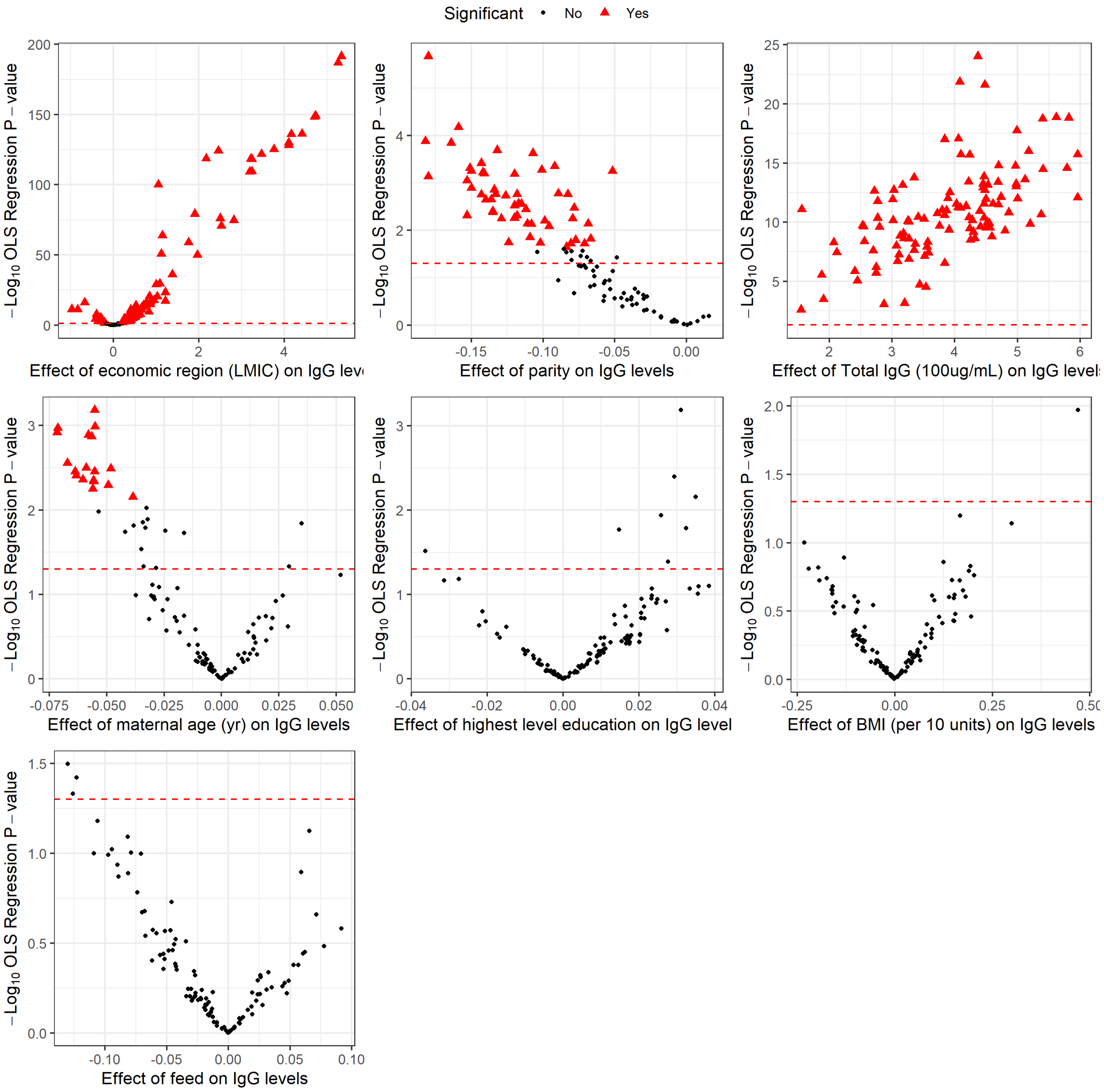

Figure S11. Effect of maternal characteristics on specific IgG antibody concentrations. The volcano plots show the effect estimates from ordinary least squares (OLS) regression models of each reactive antigen on the multipathogen protein microarray (points). The x-axis shows regression coefficients, and the y-axis shows the inverse $\log_{10}$ p-values. The red dashed line is drawn at an unadjusted p = 0.05. Antigens with signficant IgG and predictor associations after correction for the false discovery rate are shown in red triangles. Each multivariable model was fit to cohorts with complete predictor data sets or minimal missing values (see Methods). For reference, each -1.0 unit effect estimate represents a 50% decrease in IgG concentrations, and each 1.0 unit effect estimate represents a doubling of IgG concentrations.

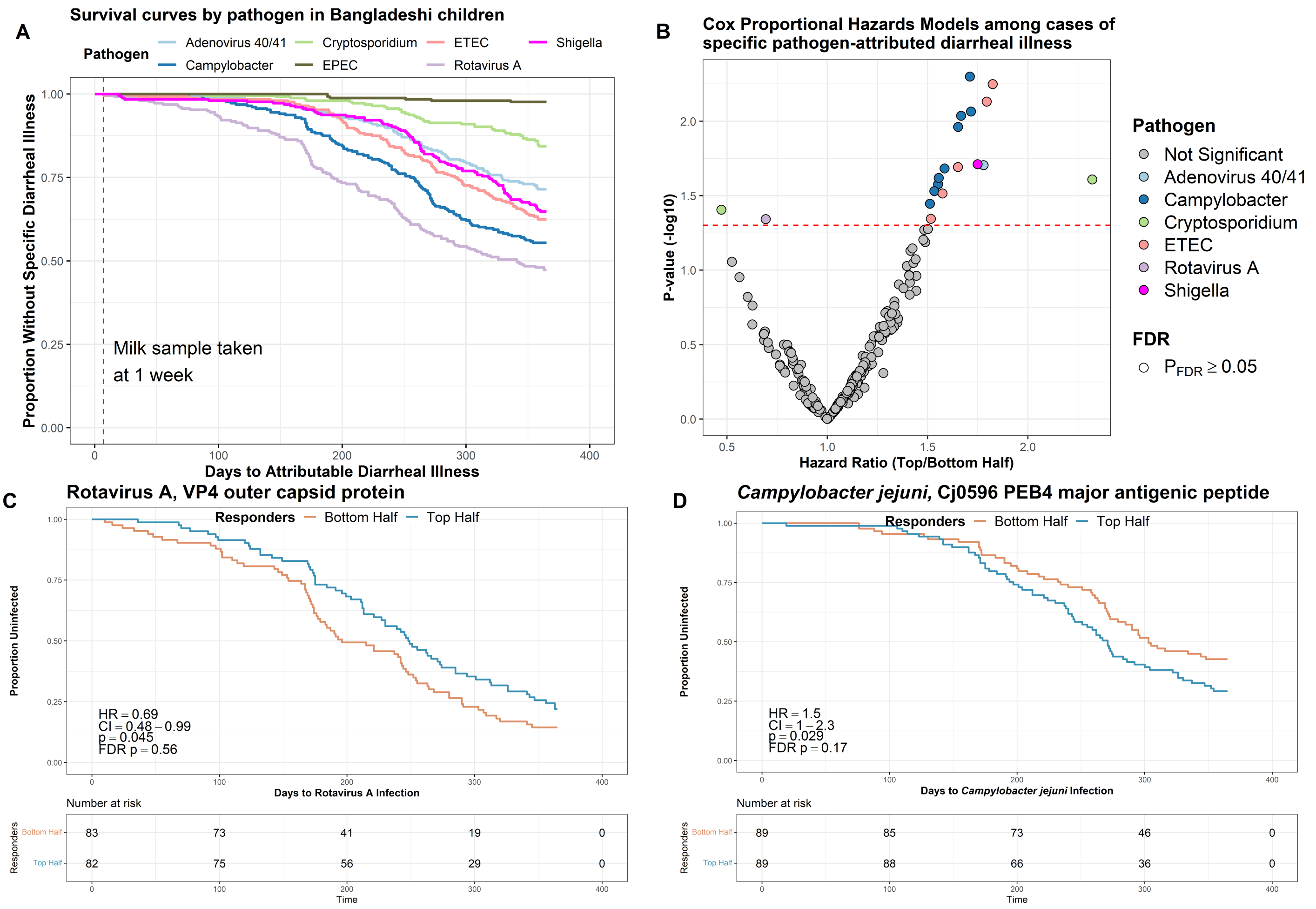

Figure S12. Association of pathogen-specific human milk IgA with disease outcomes in breastfed infants with confirmed pathogen-specific infections by PCR. (**A**) The Kaplan-Meier plot shows the survival curves of 256 infants from the PROVIDE cohort, followed from birth to 1 year for having an attributable fraction score (AFE) indicating causality of enteric pathogen on diarrheal illness. The vertical dashed red line represents the human milk sampling time point at approximately 7 days postpartum. (**B**) The volcano plot shows the hazard ratio of subjects divided into the top and bottom halves of IgA responses for each antigen. Only antigens where IgA responses were positive in at least 10% of the study women are shown. For each specific antigen, IgA responses in mothers with infants that subsequently had pathogen-specific PCR-confirmed infection were divided by the median, with the bottom and top halves included in Cox proportional hazards models on time to pathogen-attributed diarrheal illness with the corresponding pathogen. For raw p-values < 0.05, antigens were colored by pathogen (otherwise grey), and for false discovery rate (FDR)-adjusted p-values < 0.05, antigens were plotted as triangles (none identified in these models). Values below 1.0 represent lower risk rate of infection in the top half group compared with the bottom half group. (**C-D**) The Kaplan-Meier plots show the Rotavirus A VP4 outer capsid protein (**C**) and *Campylobacter jejuni* PEB4 protein (**D**) from Cox proportional hazards models of time to pathogen-attributed enteric illness among the cases of specific infections detected by PCR in stool samples of infants. The top and bottom halves of IgA responders for each antigen are shown, corresponding to the samples included in the models shown in the volcano plot (**B**). The model coefficient (β), confidence interval (CI), log-rank test p-value and adjusted p-value (BH) are shown in the bottom left. The table below each graphic shows the number at risk during 100-day intervals. The Rotavirus A non-structural protein 3 is representative of antibodies associated with longer time to infection, while the *C. jejuni* flagellar L-ring protein FlgH protein (Cj0687) represents antibodies associated with a shorter time to infection.

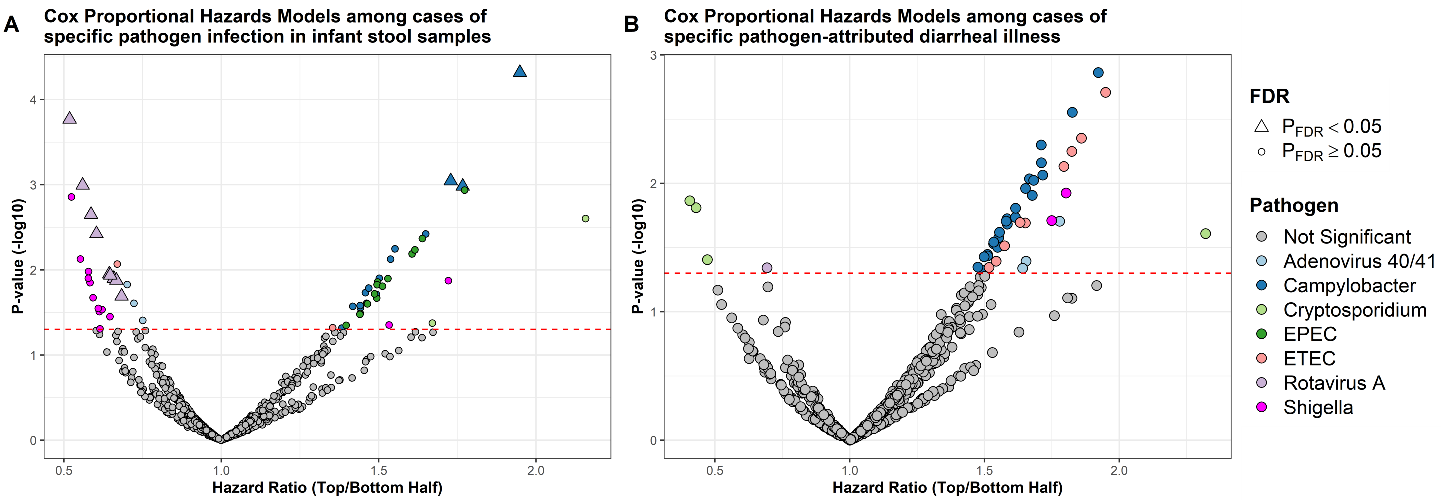

Figure S13. Association of human milk IgA with confirmed pathogen-specific infection and diarrheal disease in breastfed infants, including all antigens. (**A-B**) Hazard ratio of subjects from the PROVIDE cohort divided into the top and bottom halves of IgA responses for each antigen, irrespective of IgA positivity rate among women. IgA responses in mothers with infants that subsequently had pathogen-specific infection were included in multivariable Cox proportional hazards models on time to first infection (**A**) or time to first pathogen-attributable diarrheal illness (**B**) with the corresponding pathogen. Values below 1.0 represent lower risk of infection or disease in the top half group compared with the bottom half group. For raw p-values < 0.05, antigens were colored (otherwise grey), false discovery rate (FDR) adjusted p-values < 0.05 were plotted as triangles. P_{FDR}: multivariable Cox proportional hazards model p-value adjusted for the False Discovery Rate.

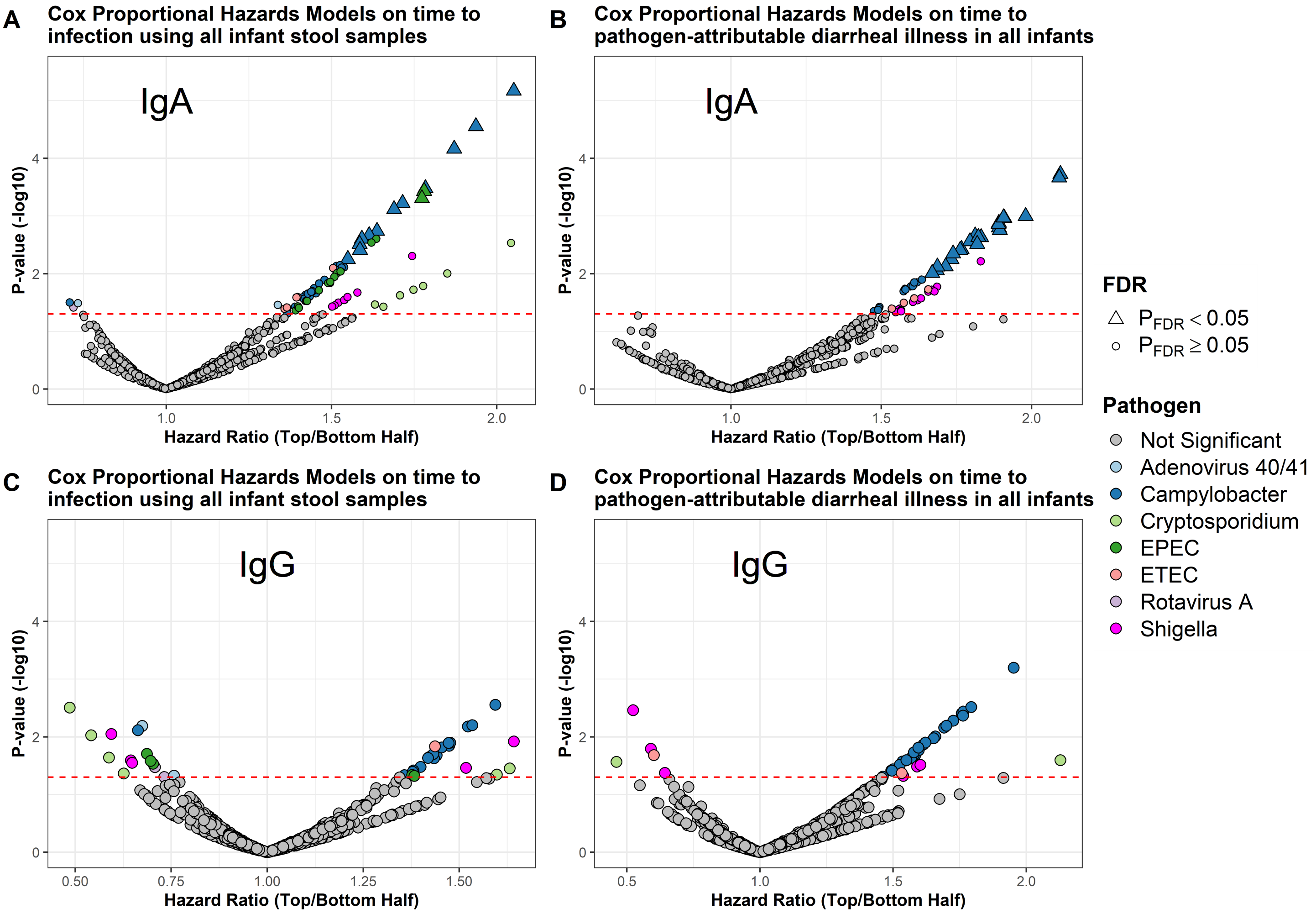

Figure S14. Association of pathogen-specific human milk IgA and IgG with infection and disease outcomes in breastfed infants. (**A-D**) The volcano plots show the hazard ratio of all 256 Bangladeshi mothers divided into the top and bottom halves of IgA responses for each antigen. For each specific antigen, IgA and IgG responses in all mothers were divided by the median, with the bottom and top halves included in Cox proportional hazards models on time to infection confirmed by PCR or pathogen-attributed diarrheal illness with the corresponding pathogen. For raw p-values < 0.05, antigens were colored by pathogen (otherwise grey), and for false discovery rate (FDR)-adjusted p-values < 0.05, antigens were plotted as triangles. Values below 1.0 represent lower risk rate of infection in the top half group compared with the bottom half group. (**A**) Human milk IgA associated with time to specific pathogen infection confirmed by PCR; (**B**) Human milk IgA associated with time to specific pathogen-attributed diarreheal illness; (**C**) Human milk IgG associated with time to specific pathogen infection confirmed by PCR; (**D**) Human milk IgG associated with time to specific pathogen-attributed diarrheal illness. P_{FDR}: multivariable Cox proportional hazards model p-value adjusted for the False Discovery Rate.

Supplemental Tables

Table S1. Study, subject and sample characteristics for human milk and maternal serum assessed.

Country (study), [total N samples available]	Country Economic Category	Sample Collection Time Point(s), Weeks [N]	Age, Years (SEM) [N]	Parity (IQR) [N]	BMI, kg/m^2(SEM) [N]	Community	Infant Outcomes Used
**Human milk**							
**Bangladesh (MDIG/MDARI)** [1, 2] **[834]**	LMIC	13 [246]	23.3 (0.3) [246]	2 (1-2) [246]	23.6 (0.3) [242]	Urban, peri-urban	Respiratory, in the first 6 months*
**Bangladesh (PROVIDE)**[3, 4] **[700]**	LMIC	1 [256]	24.7 (0.3) [256]	2 (1-3) [256]	21.5 (0.2) [256]	Urban	Enteric, at 1 year*
**Pakistan (PrePY)** [5] **[156]**	LMIC	0 [49], 6 [49], 14 [49]	26.5 (1) [44]	-	30.2 (2.7) [21]	Urban peri-urban	-
**Peru (MAL-ED)**[6] **[105]**	LMIC	0-4 [36], 8-9 [34], 13-18 [35]	24.4 (1.1) [35], 24.6 (1.1) [34], 24.1 (1.1) [35]	2 (1-3) [35], 2 (1-3.75) [34], 2 (1-3.5) [35]	24 (0.7) [35], 23.8 (0.7) [34], 23.6 (0.6) [35]	Peri-urban	-
**Rochester, NY, U.S.A. (ZOOM piloturban arm)**[8] **[23]**	HIC	6 [23]	32.3 (0.8) [21]	2 (1-3) [19]	26.5 (1.5) [17]	Urban, suburban	-
**Finland (Flora)**[9] **[300]**	HIC	0 [15], 12 [85]	-	1 (1-2) [15], 1 (1-2) [85]	-	Urban, suburban	-
Bangladesh (CBS) *[20-22]*	*LMIC*	*4 [144]*	*23.7 (0.6) [144]*	-	*22.3 (0.3) [144]*	*Urban*	*Enteric, at 1 year**
**Matched serum**							
**Bangladesh (MDIG)**[1]	LMIC	12 [60]	22. 8 (0.5) [60]	2 (1-2) [60]	23.2 (0.5) [58]	Urban, peri-urban	-
**Peru (MAL-ED)**[6]	LMIC	6 [34]	24.3 (1.1) [34]	2 (1-3.75) [34]	23.8 (0.7) [34]	Periurban	-

LMIC, low-middle income country; HIC, high income country; SEM, standard error of the mean; IQR, interquartile range; "-", data not available. Race and ethnicity is not data is not available. Means are presented with SEM, and Medians are presented with IQR. Time point 0 indicates colostrumAll the samples available from the smaller studies were assayed at desired time points, and for larger cohorts (200 or more samples), approximately one-third of the samples were selected as representative of the whole cohort or based on the case-control design to include positive and negative infant infectious outcomes where available*. Italics indicates the validation cohort.

Table S2. Linear mixed effects regression model effect estimates of biological and environmental predictors of IgG antibody concentrations against all pathogen proteins on the multipathogen protein microarray in mature human milk.

LMER Predictor	Effect Estimate	Std. Err.	t-value	Adj. p-value	Cohorts in model [N]
Economic Region (LMIC/HIC)[1]	0.93	0.037	25.0	**8.4e-83**	U.S. [19], Finland [85], Peru [35], Bangladesh (MDIG) [246]
Parity[1]	-0.08	0.015	-5.19	**3.0e-7**	U.S. [19], Finland [85], Peru [35], Bangladesh (MDIG) [246]
Human milk total IgG (per 100 mg/mL)[1]	3.87	0.23	16.8	**4.5e-48**	U.S. [19], Finland [85], Peru [35], Bangladesh (MDIG) [246]
BMI (per 10 units)[2]	-0.01	0.047	-0.25	0.8	U.S. [15], Peru [35], Bangladesh (MDIG) [242]
LMIC only					
Maternal age (yr)[3]	-0.014	0.006	-2.28	0.054	Peru [35], Bangladesh (MDIG) [242]
Highest education level[3]	0.008	0.006	1.34	0.29	Peru [35], Bangladesh (MDIG) [242]
Feed[4]	-0.02	0.021	-0.81	0.41	Bangladesh (MDIG) [246]

For reference, each -1.0 unit effect estimate represents a 50% decrease in IgG concentrations, and each 1.0 unit effect estimate represents a doubling of IgG concentrations.

[1] Predictors included in multivariable model: economic region, parity and human milk total IgA

[2] Predictors included in multivariable model: BMI, economic region, parity and human milk total IgA

[3] Predictors included in multivariable model: maternal age, highest education level, BMI, cohort, parity and human milk total IgA

[4] Predictors included in multivariable model: infant feeding pattern, maternal age, highest education level, parity and human milk total IgA

1. Roth DE, Gernand AD, Morris SK, Pezzack B, Islam MM, Dimitris MC, Shanta SS, Zlotkin SH, Willan AR, Ahmed T, Shah PS, Murphy KE, Weksberg R, Choufani S, Shah R, Al Mahmud A. Maternal vitamin D supplementation during pregnancy and lactation to promote infant growth in Dhaka, Bangladesh (MDIG trial): study protocol for a randomized controlled trial. Trials. 2015;16:300. Epub 2015/07/15. doi: 10.1186/s13063-015-0825-8. PubMed PMID: 26169781; PMCID: PMC4499946.

2. Morris SK, Pell LG, Rahman MZ, Dimitris MC, Mahmud A, Islam MM, Ahmed T, Pullenayegum E, Kashem T, Shanta SS, Gubbay J, Papp E, Science M, Zlotkin S, Roth DE. Maternal vitamin D supplementation during pregnancy and lactation to prevent acute respiratory infections in infancy in Dhaka, Bangladesh (MDARI trial): protocol for a prospective cohort study nested within a randomized controlled trial. BMC pregnancy and childbirth. 2016;16(1):309. Epub 2016/10/16. doi: 10.1186/s12884-016-1103-9. PubMed PMID: 27737646; PMCID: PMC5064894.

3. Naylor C, Lu M, Haque R, Mondal D, Buonomo E, Nayak U, Mychaleckyj JC, Kirkpatrick B, Colgate R, Carmolli M, Dickson D, van der Klis F, Weldon W, Steven Oberste M, teams Ps, Ma JZ, Petri WA, Jr. Environmental Enteropathy, Oral Vaccine Failure and Growth Faltering in Infants in Bangladesh. EBioMedicine. 2015;2(11):1759-66. Epub 2016/02/13. doi: 10.1016/j.ebiom.2015.09.036. PubMed PMID: 26870801; PMCID: PMC4740306.
4. Kirkpatrick BD, Colgate ER, Mychaleckyj JC, Haque R, Dickson DM, Carmolli MP, Nayak U, Taniuchi M, Naylor C, Qadri F, Ma JZ, Alam M, Walsh MC, Diehl SA, Teams PS, Petri WA, Jr. The "Performance of Rotavirus and Oral Polio Vaccines in Developing Countries" (PROVIDE) study: description of methods of an interventional study designed to explore complex biologic problems. Am J Trop Med Hyg. 2015;92(4):744-51. Epub 2015/02/26. doi: 10.4269/ajtmh.14-0518. PubMed PMID: 25711607; PMCID: PMC4385767.
5. Omer SB, Kazi AM, Bednarczyk RA, Allen KE, Quinn CP, Aziz F, Sial K, Phadke VK, Tondella ML, Williams MM, Orenstein WA, Ali SA. Epidemiology of Pertussis Among Young Pakistani Infants: A Community-Based Prospective Surveillance Study. Clin Infect Dis. 2016;63(suppl 4):S148-S53. Epub 2016/11/14. doi: 10.1093/cid/ciw561. PubMed PMID: 27838667; PMCID: PMC5106628.
6. Investigators M-EN. The MAL-ED study: a multinational and multidisciplinary approach to understand the relationship between enteric pathogens, malnutrition, gut physiology, physical growth, cognitive development, and immune responses in infants and children up to 2 years of age in resource-poor environments. Clin Infect Dis. 2014;59 Suppl 4:S193-206. Epub 2014/10/12. doi: 10.1093/cid/ciu653. PubMed PMID: 25305287.
7. Seppo AE, Bu K, Jumabaeva M, Thakar J, Choudhury RA, Yonemitsu C, Bode L, Martina CA, Allen M, Tamburini S, Piras E, Wallach DS, Looney RJ, Clemente JC, Jarvinen KM. Infant gut microbiome is enriched with Bifidobacterium longum ssp. infantis in Old Order Mennonites with traditional farming lifestyle. Allergy. 2021. Epub 2021/04/28. doi: 10.1111/all.14877. PubMed PMID: 33905556.
8. Seppo AE, Choudhury R, Pizzarello C, Palli R, Fridy S, Rajani PS, Stern J, Martina C, Yonemitsu C, Bode L, Bu K, Tamburini S, Piras E, Wallach DS, Allen M, Looney RJ, Clemente JC, Thakar J, Jarvinen KM. Traditional Farming Lifestyle in Old Older Mennonites Modulates Human Milk Composition. Front Immunol. 2021;12:741513. Epub 2021/10/29. doi: 10.3389/fimmu.2021.741513. PubMed PMID: 34707611; PMCID: PMC8545059.
9. Kukkonen K, Savilahti E, Haahtela T, Juntunen-Backman K, Korpela R, Poussa T, Tuure T, Kuitunen M. Probiotics and prebiotic galacto-oligosaccharides in the prevention of allergic diseases: a randomized, double-blind, placebo-controlled trial. J Allergy Clin Immunol. 2007;119(1):192-8. doi: 10.1016/j.jaci.2006.09.009. PubMed PMID: 17208601.
10. McGuire MK, Randall AZ, Seppo AE, Jarvinen KM, Meehan CL, Gindola D, Williams JE, Sellen DW, Kamau-Mbuthia EW, Kamundia EW, Mbugua S, Moore SE, Prentice AM, Foster JA, Otoo GE, Rodriguez JM, Pareja RG, Bode L, McGuire MA, Campo JJ. Multipathogen Analysis of IgA and IgG Antigen Specificity for Selected Pathogens in Milk Produced by Women From Diverse Geographical Regions: The INSPIRE Study. Front Immunol. 2020;11:614372. Epub 2021/03/02. doi: 10.3389/fimmu.2020.614372. PubMed PMID: 33643297; PMCID: PMC7905217.
11. Krogh A, Larsson B, von Heijne G, Sonnhammer EL. Predicting transmembrane protein topology with a hidden Markov model: application to complete genomes. J Mol Biol. 2001;305(3):567-80. doi: 10.1006/jmbi.2000.4315. PubMed PMID: 11152613.
12. Almagro Armenteros JJ, Tsirigos KD, Sonderby CK, Petersen TN, Winther O, Brunak S, von Heijne G, Nielsen H. SignalP 5.0 improves signal peptide predictions using deep neural networks. Nat Biotechnol. 2019;37(4):420-3. Epub 20190218. doi: 10.1038/s41587-019-0036-z. PubMed PMID: 30778233.
13. Yu NY, Wagner JR, Laird MR, Melli G, Rey S, Lo R, Dao P, Sahinalp SC, Ester M, Foster LJ, Brinkman FS. PSORTb 3.0: improved protein subcellular localization prediction with refined localization subcategories and predictive capabilities for all prokaryotes. Bioinformatics. 2010;26(13):1608-15. Epub 20100513. doi: 10.1093/bioinformatics/btq249. PubMed PMID: 20472543; PMCID: PMC2887053.
14. Marchler-Bauer A, Derbyshire MK, Gonzales NR, Lu S, Chitsaz F, Geer LY, Geer RC, He J, Gwadz M, Hurwitz DI, Lanczycki CJ, Lu F, Marchler GH, Song JS, Thanki N, Wang Z, Yamashita RA, Zhang D, Zheng C, Bryant SH. CDD: NCBI's conserved domain database. Nucleic Acids Res. 2015;43(Database issue):D222-6. Epub 20141120. doi: 10.1093/nar/gku1221. PubMed PMID: 25414356; PMCID: PMC4383992.
15. Finn RD, Coggill P, Eberhardt RY, Eddy SR, Mistry J, Mitchell AL, Potter SC, Punta M, Qureshi M, Sangrador-Vegas A, Salazar GA, Tate J, Bateman A. The Pfam protein families database: towards a more sustainable future. Nucleic Acids Res. 2016;44(D1):D279-85. Epub 20151215. doi: 10.1093/nar/gkv1344. PubMed PMID: 26673716; PMCID: PMC4702930.
16. Davies DH, Liang X, Hernandez JE, Randall A, Hirst S, Mu Y, Romero KM, Nguyen TT, Kalantari-Dehaghi M, Crotty S, Baldi P, Villarreal LP, Felgner PL. Profiling the humoral immune response to infection by using proteome microarrays:

high-throughput vaccine and diagnostic antigen discovery. Proc Natl Acad Sci U S A. 2005;102(3):547-52. Epub 2005/01/14. doi: 10.1073/pnas.0408782102. PubMed PMID: 15647345; PMCID: PMC545576.
17. Lex A, Gehlenborg N, Strobelt H, Vuillemot R, Pfister H. UpSet: Visualization of Intersecting Sets. IEEE Trans Vis Comput Graph. 2014;20(12):1983-92. Epub 2015/09/12. doi: 10.1109/TVCG.2014.2346248. PubMed PMID: 26356912; PMCID: PMC4720993.
18. Bates D, Mächler M, Bolker B, Walker S. Fitting Linear Mixed-Effects Models Using lme4. Journal of Statistical Software. 2015;67(1):1 - 48. doi: 10.18637/jss.v067.i01.
19. Benjamini Y, Hochberg Y. Controlling the false discovery rate: a practical and powerful approach to multiple testing. Journal of the Royal Statistical Society: Series B (Methodological). 1995;57(1):289-300.
20. Kabir M, Alam M, Nayak U, Arju T, Hossain B, Tarannum R, Khatun A, White JA, Ma JZ, Haque R, Petri WA, Jr., Gilchrist CA. Nonsterile immunity to cryptosporidiosis in infants is associated with mucosal IgA against the sporozoite and protection from malnutrition. PLoS Pathog. 2021;17(6):e1009445. Epub 20210628. doi: 10.1371/journal.ppat.1009445. PubMed PMID: 34181697; PMCID: PMC8270466.
21. Steiner KL, Kabir M, Hossain B, Gilchrist CA, Ma JZ, Ahmed T, Faruque ASG, Haque R, Petri WA. Delayed Time to Cryptosporidiosis in Bangladeshi Children is Associated with Greater Fecal IgA against Two Sporozoite-Expressed Antigens. Am J Trop Med Hyg. 2021;104(1):229-32. doi: 10.4269/ajtmh.20-0657. PubMed PMID: 33078702; PMCID: PMC7790099.
22. Steiner KL, Kabir M, Priest JW, Hossain B, Gilchrist CA, Cook H, Ma JZ, Korpe PS, Ahmed T, Faruque ASG, Haque R, Petri WA. Fecal Immunoglobulin A Against a Sporozoite Antigen at 12 Months Is Associated With Delayed Time to Subsequent Cryptosporidiosis in Urban Bangladesh: A Prospective Cohort Study. Clin Infect Dis. 2020;70(2):323-6. doi: 10.1093/cid/ciz430. PubMed PMID: 31131855; PMCID: PMC6938969.
